# The role of epigenetic modifications in hematological cancers

**DOI:** 10.3389/fonc.2026.1744018

**Published:** 2026-02-05

**Authors:** Jovana Ilic, Anna Bold, Stefan Knop

**Affiliations:** Department of Hematology and Medical Oncology, Paracelsus Medical University, Nuremberg, Germany

**Keywords:** chromatin remodeling, DNA methylation, epigenetics, hematological cancers, histone modifications

## Abstract

Epigenetic regulation of gene expression entails DNA methylation and histone modifications, which orchestrate chromatin structure and transcriptional activity. Aberrant regulation of these mechanisms contributes to the development and progression of hematological cancers. Mutations in enzymes mediating DNA methylation and histone modification patterns reshape chromatin structure, influencing transcriptional profiles, thus promoting oncogenesis, clonal evolution and drug resistance. This review focuses on significant alterations in DNA methylation patterns, mutations and changes in expression of histone modifying enzymes and chromatin remodeling complexes described in leukemias, lymphomas and multiple myeloma. We summarize the most prominent changes in epigenetic mediators and their impact on transcriptional activity and oncogenesis.

## 1 Introduction

Epigenetics is a broad term that encompasses several different cellular mechanisms that alter gene expression independently of the DNA sequence. Epigenetic regulation of gene expression facilitated the evolution of multicellular organisms, enabling a dynamic control of cellular processes and differentiation necessary for diverse tissue formation and complexity (1). Epigenetic processes include DNA methylation, histone modifications, and chromatin remodeling. These mechanisms allow swift cellular responses to ever-changing cellular signals through alterations in DNA conformation and its transcriptional availability (2). In addition, regulatory non-coding RNAs (ncRNAs) are non-protein coding transcripts which regulate transcription, translation, splicing, and chromatin remodeling, influencing all cellular functions. Even though ncRNAs are closely involved in epigenetic processes, they are not an epigenetic mechanism per se. Given the vast diversity of their sizes, roles and mechanisms of action, they could be considered a distinct entity and will therefore not be discussed in this review (3).This review will focus on classical epigenetic mechanisms (DNA methylation, histone modifications and chromatin remodeling complexes) to maintain a defined scope.

Hematological cancers originate from cells generated via hematopoiesis and include leukemias, lymphomas, and multiple myeloma (4). Hematopoiesis is a closely regulated process of generating new components of circulating blood cells and the immune system from a common hematopoietic stem cell progenitor (5). To date, many epigenetic factors have been identified to play a role in this complex, multi-stage differentiation process. Cancer cells from the same individuals differ in their phenotype and functionality, generating a heterogeneous population, and are thus difficult to therapeutically target at once. Therefore, it has become clear that epigenetic involvement plays a crucial role in the development, maintenance, and resistance of malignancies. In the context of hematological malignancies, aberrant DNA methylation and mutations in histone modification and chromatin remodeling enzymes are observed frequently (6). Unraveling the epigenetic patterns of these cancers is essential for their deeper understanding and improved clinical outcomes.

## 2 DNA methylation in hematological malignancies

DNA methylation includes the addition of a methyl group to cytosine, within the cytosine-guanine dinucleotides (CpG). It is conveyed by DNA methyltransferase (DNMT) enzymes and it is widely spread out over the whole genome (7). The DNA methylation pattern at cytosine residues within CpG sequences is established during early blood cell development and inherited through cell divisions. Many transgenic regions in the genome, including transposons, are regulated via methylation. DNMT1 is mainly involved in the methylation maintenance during replication, while DNMT3, especially DNMT3A, and DNMT3B, are responsible for the *de novo* methylation in the genome (8). Widespread genomic hypomethylation is often observed in cancer, coinciding with the activity of mobile genetic elements and genomic instability, contributing to higher mutagenicity. In addition, many tumor suppressor genes and microRNAs (miRNAs) are silenced by promoter hypermethylation in cancer cells (9, 10). Both *DNMT1* and *DNMT3* are strongly linked to hematological malignancies. *DNMT1* is frequently overexpressed in cancer cells and seems to promote tumor development, whereas *DNMT3A* is regarded as tumor suppressive, often downregulated and mutated in hematological cancers. *DNMT3B* exhibits a dual role in hematological malignancies, with data confirming both its oncogenic and tumor suppressive functions (11, 12). On the other hand, members of the ten-eleven translocation methylcytosine dioxygenase (TET) family of enzymes are involved in DNA demethylation and are often found to be mutated in hematological malignancies as well (13). TET enzymes oxidize 5-methylcytosine (5mC) to 5-hydroxymethylcytosine (5hmC), an intermediate step in the DNA demethylation process. TET generated intermediates are recognized and excised by thymine-DNA glycosylase (TDG), and cytosine is restored through the base excision repair (BER) mechanism (14). A prominent member of the TET family, *TET2*, is highly expressed in hematopoietic stem and progenitor cells and regulates self-renewal and differentiation. This function correlates to its role in hematological cancers originating from immature progenitors (15).

**Figure 1:**
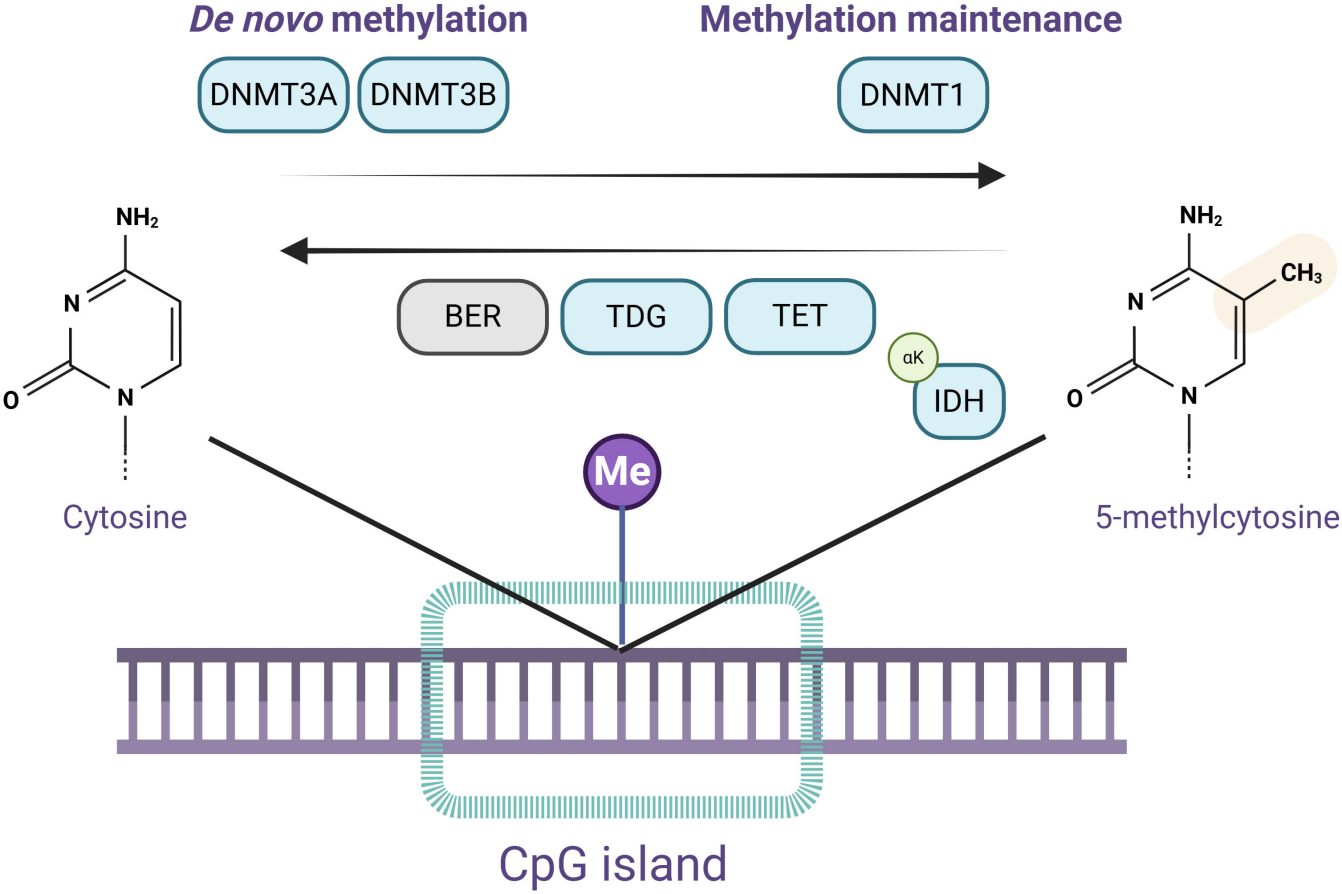
Overview of DNA methylation and demethylation pathways. Schematic representation of DNA (de)methylation pathways within the CpG islands. DNA methyltransferases DNMT3A and DNMT3B catalyze *de novo* methylation, forming 5-methylcytosine. DNMT1 is responsible for methylation maintenance of newly synthesized DNA strands during replication. Active DNA demethylation is carried out by Ten–Eleven Translocation (TET) enzymes, which oxidize 5-methylcytosine into intermediates recognized and excised by Thymine DNA Glycosylase (TDG). The excised nucleotide is repaired by the base excision repair (BER) machinery, restoring cytosine. Isocitrate Dehydrogenase (IDH) enzymes produce αKG, a cofactor necessary for TET activity. Image created in Biorender.

An overview of DNA methylation is provided in **Figure 1**.

### 2.1 Mutations in DNA methyltransferase enzymes

To date, *DNMT3A* mutations have been identified in most hematological malignancies (16–20). For example, *DNMT3A* mutations are identified in one third of acute myeloid leukemia (AML), myeloproliferative neoplasia (MPN) and myelodysplastic syndrome (MDS) patients (21). Loss of *DNMT3A* function disrupts hematopoietic cell differentiation, favoring an expansion of hematopoietic stem cells which have the ability to self-renew (22). Self-renewing cells present a significant treatment hurdle, as they usually represent the drug-resistant cancer cell reservoir (23). Notably, hematopoietic stem cells from AML patients with *DNMT3* mutations displayed increased treatment resistance and a potential for secondary mutations and relapse, with shortened median overall survival (16, 24). The activity of DNMT3 is correlated with tumor suppression and its mutation or downregulation is considered an early driver event in leukemia and multiple myeloma (MM) development as well. In fact, the promoter of the *DNMT3A* itself was hypermethylated in MM cells, causing its reduced expression (21, 25, 26). However, *DNMT3* mutations were not correlated with their exact functions in these cancers and their fundamental consequences remained unknown.

*DNMT1* overexpression is a frequent occurrence in other hematological cancers and leads to silencing of tumor suppressor genes due to promoter hypermethylation (27, 28). For instance, in MM, increased DNMT1 protein activity downregulates the tumor suppressor cyclin-dependent kinase inhibitor 2A (p16, *CDKN2A*), leading to dysregulated proliferation (29). Similarly, the increased activity of DNMT1 was observed across leukemia and related to demethylation of its promotors (30). Functionally, this led to tumor suppressor silencing via promotor hypermethylation, such as cyclin-dependent kinase inhibitor 2B (p15, *CDKN2B*) in AML, Src Homology region 2 domain-containing Phosphatase-1 (SHP1) in chronic myeloid leukemia (CML), and Phosphatase and Tensin Homolog (PTEN) in B-cell Acute Lymphoblastic Leukemia (B-ALL) (11, 31, 32). In highly proliferating cancer cells, where DNA replication is prominent, the overexpression of *DNMT1* might initially occur as a regulatory response. Consequently, this leads to hypermethylation of cell cycle regulatory genes and promotes proliferation. *DNMT1* overexpression is associated with drug resistance, poor clinical prognosis, enhanced stem-like characteristics, and increased aggressiveness. These outcomes are a consequence of a disturbed cell cycle and hypermethylation of regulatory protein promoters (11).

The activity of DNMT3B enzymes was investigated less. There are several studies confirming their contrasting roles in hematological cancers, depending on the tumor microenvironment, catalytic activity, and the experimental system applied for its investigation. For instance, loss of DNMT3B catalytic activity caused decreased methylation of the oncogenic mesenchymal–epithelial transition factor (*Met*) promoter in mice, leading to its upregulation. In turn, this resulted in increased Signal transducer and activator of transcription 3 (STAT3) phosphorylation and lymphomagenesis (12). In humans, the *DNMT3B* promoter was methylated in some cases, causing its downregulation, which led to tumor growth and elevated stemness in AML cells (33). Taken together, these studies suggest the tumor suppressive role of DNMT3B.

Nonetheless, the oncogenic activity of DNMT3B is discussed, as the levels of this enzyme were increased in T-ALL and Burkitt’s lymphoma, coinciding with increased oncogenic activity of myelocytomatosis (*MYC*) (34). However, the increased *DNMT3B* expression may occur as a consequence of a positive feedback loop, with the goal to reduce the activity of c-Myc, indicating a tumor suppressive role. This is supported by the fact that a knockdown of *MYC* in both T-ALL and Burkitt’s lymphoma cell lines caused a downregulation of *DNMT3B* (34). No conclusive data has been acquired on alternative transcript processing as a potential underlying cause of the enzyme’s different functionality in hematological cancers. However, it is indicated that a truncated isoform of this enzyme, DNMT3B7, which is catalytically inactive and commonly found in cancer cells, contributes to *Myc*-driven lymphomagenesis in a transgenic mouse model (35). This suggests that isoform presence, rather than total DNMT3B levels, might determine the biological outcome, but this remains unexplored in human primary samples. In addition, the oncogenic role of specific enzymes can be disease-specific, since these malignancies have their own characteristics, different origins, and a variable proteomic milieu.

### 2.2 Mutations in ten-eleven translocation methylcytosine dioxygenase enzymes

Members of the TET family of enzymes, such as TET1, TET2, and TET3 are generally considered tumor suppressors in hematological cancers, since their deregulation contributes to disease development (13). *TET2* mutations are more frequent and are associated with myeloid malignancies, whereas *TET1* mutations are found in lymphoid malignancies and are not that common overall (36–38). *TET1* is often silenced epigenetically in hematological cancers, including follicular lymphoma (FL) and MM, through promoter methylation (39). In contrast, *TET2* is one of the most frequently mutated genes in myeloid malignancies:10-30% in AML, 20-30% in MDS, and up to 65% in chronic myelomonocytic leukemia (CMML) cases (38, 40). These mutations often coincide with other recurrent genetic lesions, including *DNMT3A* and isocitrate dehydrogenase 1/2 (*IDH1/2*), indicating that *TET2* loss cooperates with additional molecular events to drive leukemogenesis (41).

Despite their prevalence, no mutational hotspots have been identified in the *TET2* gene in hematological cancers, as the mutations were distributed throughout the entire coding region (42). Additionally, these mutations were not recognized in other somatic cells, suggesting that *TET2* loss of function may be a driver event in hematologic carcinogenesis (43). In AML, the loss of *TET2* is a consequence of nonsense or missense mutations and frameshift indels within the catalytic domain, resulting in impaired enzymatic activity. In murine *in vivo* models, *Tet2* loss of function resulted in elevated self-renewal of hematopoietic cells, coinciding with disturbed hematopoietic differentiation and an abnormal accumulation of immature myeloid cells (44, 45). Dysregulated differentiation directly contributes to the development of clonal hematopoiesis, which clinically manifests as increased risk for cancer progression and drug resistance, with poorer overall survival rates. In addition, *TET2* loss in hematopoietic stem cells causes an increased mutational burden, observed both in mice and myeloid malignancy patients (46). This is consistent with clinical data showing how *TET2*-mutated clones persist after treatment and lead to clonal evolution and relapse in AML patients (47). The elevated mutational load creates an environment for additional oncogenic mutations, linking *TET2* deficiency to disease development. *TET2* mutations were associated with decreased levels of 5hmC in MPN, CMML, MDS, and AML patient samples (48, 49). Reduced 5hmC levels disrupt regular gene expression patterns, favoring the survival of immature, persistent clones. This leads to therapy resistance and higher relapse rates. In mice, specific *Tet2* mutations were linked to extramedullary hematopoiesis, increased repopulation capacity, splenomegaly and elevated myelopoiesis, which could not have been compensated by the other two TETs (50, 51).

While TET2 has been extensively investigated in hematological malignancies, less is known about TET3. TET3 is involved in the maintenance of genomic stability in hematopoietic cells (52). In some lymphoid malignancies, the downregulation of *TET3* has been observed, but its precise role in carcinogenesis is not described (36).

TET loss of function contributes to hematological cancers through many synergistic mechanisms, which are not yet fully understood. For example, loss of TET proteins changes DNMT3A localization and can paradoxically contribute to DNA hypomethylation, which promotes genomic instability and disrupts hematopoiesis (53). These changes lead to aberrant self-renewal and differentiation in hematopoietic progenitors, promoting oncogenesis. Taken together, current studies indicate a correlation between TET impairment and a more stem-like behavior of cancer cells, increasing their chances of survival and leading to drug resistance and relapse.

### 2.3 Mutations in isocitrate dehydrogenase enzymes

IDH enzymes are indirectly involved in the DNA demethylation process. They mediate the conversion of isocitrate to α-ketoglutarate (αKG), a cofactor of TETs. Mutations in IDH genes result in the inhibition of TET enzyme activity which disrupts normal epigenetic regulation and cell differentiation (54). Mutated IDH enzymes mediate the production of an oncometabolite, D-2-hydroxyglutarate (2-HG), which mimics αKG and impacts TET function, influencing epigenetic regulation (55).

In AML, *IDH1* and *IDH2* mutations were recognized in about 20% of patients. This correlated with specific (and similar) methylation profiles, with a hypermethylated DNA signature (54, 56). Despite their high prevalence, *IDH1/2* mutations rarely co-occur with *TET2* mutations in AML. These mutations ultimately result in TET disfunction and seem to be mutually exclusive (57).

Besides AML, *IDH* mutations were not reported in other hematological cancers with such frequency, except for a very rare disease, angioimmunoblastic T-cell lymphoma (AITL), where they are found in up to 45% of patients (58–60). In AILT, *IDH2* mutations coincide with *TET2* and *DNMT3A* mutations in T follicular helper cells. Acting synergistically, these mutations contribute to genomic instability and oncogenesis (61). Unlike in AML, *IDH2* mutations might not be sufficient to inhibit TET activity on their own in AITL. This suggests that both *TET2* and *DNMT3A* loss is needed to disrupt the genome organization.

Since they might follow different epigenetic patterns, different hematological cancers (especially those of different origin) should be investigated separately, with the aim of identifying shared underlying principles. Taken together, identified mutations in both *DNMT*, *TET*, and *IDH* gene families highlight that the balance and patterns of DNA methylation are key distinguishing factors between healthy and neoplastic cells.

### 2.4 Specific methylation patterns correlated with hematological malignancies

Despite extensive data collected on mutations in enzymes involved in DNA (de)methylation, their functional consequences, enzymatic activity, and clinical relevance are not always defined. DNA methylation patterns, a result of these enzymatic processes, may provide deeper insights into the development and maintenance of hematological cancers. Global hypomethylation and focal hypermethylation of DNA are well recognized in hematological malignancies.

In myeloid neoplasms (AML and MDS), specific promoters of genes involved in the inhibition of Wingless and Int-1 (WNT) and mitogen activated protein kinase (MAPK) signaling pathways were hypermethylated, compared with healthy donor CD34^+^ bone marrow cells. This resulted in the overactivation of these pathways, sustaining an immature cellular phenotype, consistent with the impaired differentiation linked to the development of these cancers (62). The progression of MDS to AML is believed to be driven by aberrant methylation of tumor suppressor genes (63). Accordingly, the identification of DNA methylation profiles, or epitypes, might support AML patient stratification as it provides insights into disease pathogenesis and could dictate therapeutic strategies (64). For example, *Kelly* et al. identified specific CpG island DNA methylation phenotypes in AML that correlated with disease-free survival (65). Currently, DNA methylation-based bioinformatics tools are being developed to improve diagnostic predictions and early detection in patients with AML and B-cell Non-Hodgkin Lymphoma (66, 67).

Similarly, in MM, DNA methylation patterns correlate with the stage of the disease and can be used as biomarkers (26). MM can be preceded by a non-malignant condition called monoclonal gammopathy of undetermined significance (MGUS). This transition coincides with global hypomethylation, focal hypermethylation, and altered *DNMT3* expression (26, 68). Extensive hypermethylation of B-cell specific enhancers and cyclin-dependent kinase inhibitors, as well as Wnt antagonists leads to a less differentiated, more aggressive stem-like cancer cell phenotype (69, 70). Comparable to AML, hypermethylation of promoter regions in Wnt signaling pathway inhibitor genes led to an overactivation of Wnt signaling in both MM cell lines and primary MM samples, which correlates with a less differentiated MM phenotype (71, 72). This is another example of DNA methylation patterns correlating with stem-like traits of cancer cells, directly affecting their drug response and relapse. To that matter, demethylation of the ATP-binding cassette transporter G2 (*ABCG2*) promoter is treatment inducible in MM cells and causes its overexpression, increasing drug resistance (73).

In lymphoid malignancies such as ALL, certain tumor suppressor gene promoters were methylated. However, these methylation patterns were often stochastic and contributed to the heterogeneity of the disease (74, 75).

In contrast, in Chronic Lymphocytic Leukemia (CLL) patients, no specific methylation profiles were correlated with treatment response, likely due to its indolent biology (76).

Most DNA methylation alterations in hematological malignancies follow similar patterns, which ultimately lead to increased oncogenic activity and tumor suppressor inactivation. However, this conclusion might stem from research bias, as most studies focus on specific loci methylomes, particularly within the coding regions, driven by an interest in the functional consequences of the methylation. Not much research has been conducted in recent years on this topic, although additional studies and analyses with the data already available might provide new insights due to the rise in the simplicity and computational power of (bio)informatics tools.

## 3 Histone modifications in hematological malignancies

**Figure 2:**
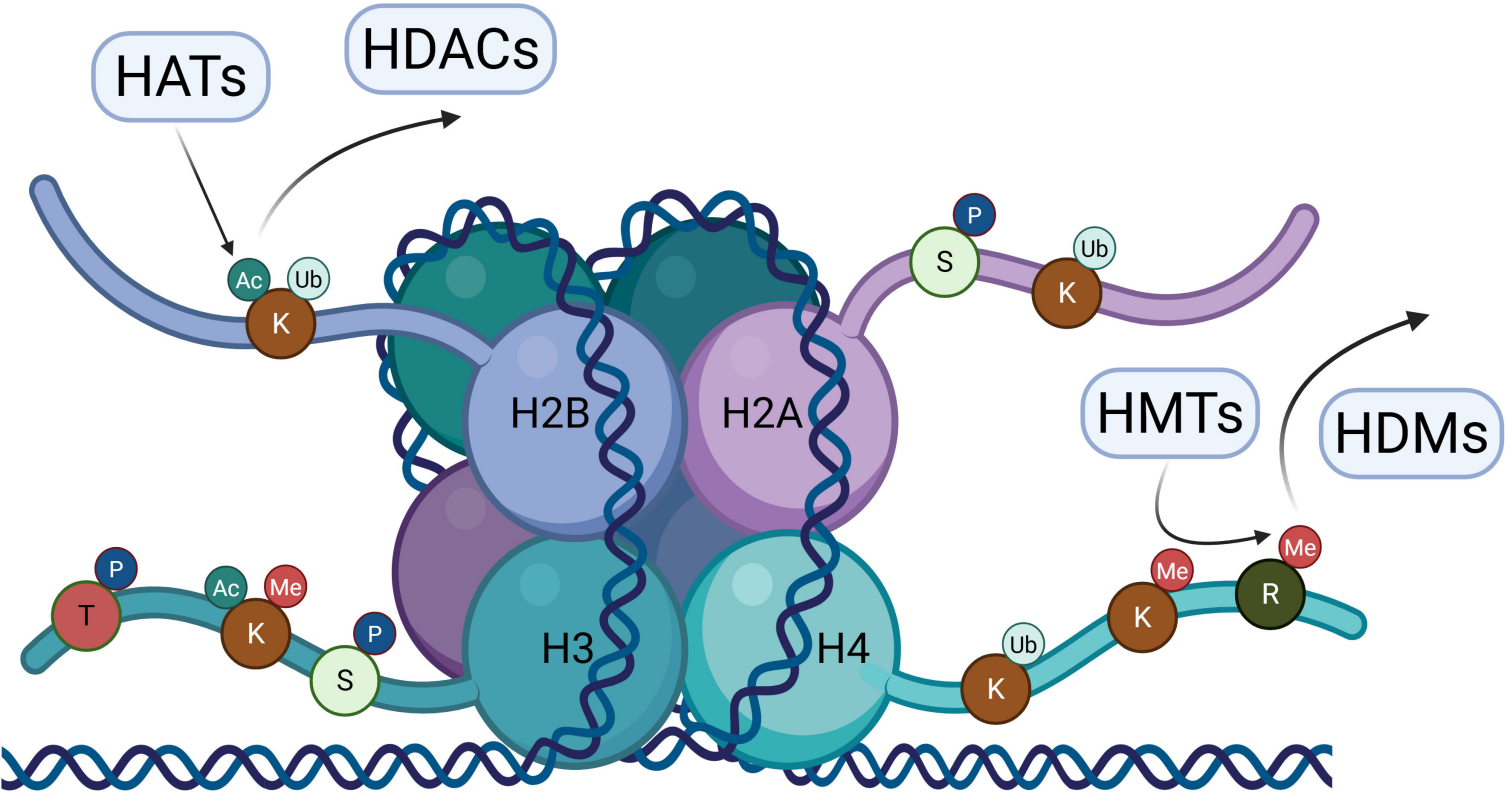
Histone modification landscape and major enzymes. Schematic representation of a nucleosome composed of the histone H2A, H2B, H3 and H4 octamer with DNA wrapped around it. Amino acids located on the N-terminal histone tails such as lysine (K), serine (S), threonine (T) and arginine (R) are modified post-translationally. The most common histone modifications, such as methylation (Me), acetylation (Ac), phosphorylation (P) and ubiquitination (Ub) are shown in the image. Histone methyltransferases (HMTs) catalyze the addition of methyl groups on lysine or arginine residues, while histone demethylases (HDMs) catalyze their removal. Histone acetyltransferases (HATs) and deacetylases (HDACs) are involved in the addition or removal of acetyl groups from lysine residues. These dynamic combinations of histone modifications regulate chromatin structure and gene expression by modulating DNA accessibility and downstream protein recruitment. Image created in Biorender.

Histones are proteins that provide structural support to DNA. Together with histones, DNA forms nucleosomes – the basic building units of chromatin. Histone modifications are posttranslational changes of histones including phosphorylation, ubiquitination, acetylation, methylation, sumoylation, etc. (**Figure 2**). Methylation and acetylation are most commonly investigated in cancer and their functions are well described (77). Ubiquitination of histones occurs as a regulatory signal and seldom as a mark for protein degradation, as there is no sequential addition of this peptide (78). Specific combinations of histone modifications comprise the histone code, which determines chromatin structure and the recruitment of downstream effector proteins. The crosstalk between DNA and histone modifications directs the interactions with protein complexes involved in replication, transcription, DNA repair and chromatin structure. Thus, modification of histones is responsible for gene expression regulation and genome stability. Disruption of the balance of histone modifications is involved in tumorigenesis, as it leads to dysregulated gene expression and genome instability (77).

### 3.1 Histone methylation in hematological malignancies

Methylation of histones is mediated by histone methyltransferase enzymes (HMTs) and histone demethylases (HDMs). It entails the addition or removal of methyl groups to the lysine or arginine residues of (mostly H3 and H4) histone proteins. Histone methylation is considered to repress transcription, but can also be involved in transcription activation, depending on the methylated histone, degree of methylation and the amino acid (position) (79, 80).

Mutations in histone methylation proteins often affect stemness and differentiation in hematological malignancies and are described extensively in cancers arising from immature progenitors, such as AML and ALL. Rearrangements of the lysine methyltransferase 2A (*KMT2A*) gene in cancer cells were recognized a while ago as drivers of ALL, particularly in children (81). *KMT2A* translocation results in aberrant fusion proteins and occurs in mixed lineage leukemia (MLL), a subtype of the disease characterized by this mutation (82). *KMT2A* is essential for hematopoietic stem cell development and maintenance, which explains its role in immature blood cancers (83). Mutations in this gene affect chromatin structure and gene expression regulation and are directly involved in ALL pathogenesis via H3 lysine methylation (H3K4) (82). H3K4 methylation in the promoter region facilitates the transcription of Homeobox protein Hox-A9 (*HOXA9*) in both ALL and AML (84, 85),. Upregulation of *HOXA9* promotes pathological progenitor self-renewal and impaired differentiation, which can lead to more aggressive tumor behavior and poor clinical outcomes (86, 87). *KMT2A* rearrangements can also be regarded as a prognostic marker in ALL and AML. In AML, up to 10% of patients exhibit *KMT2A* translocations, resulting in aberrant fusion proteins, and their frequency can increase upon treatment (88). This post-therapy increase in *KMT2A*-mutated clones supports the idea that these mutations govern a persistent, self-renewing subpopulation capable of drug resistance and tumor repopulation.

Besides KMT2A, additional histone methylation enzymes are involved in the development and persistence of AML. For instance, dysregulation of Polycomb repressive complex 2 (PRC2) histone methylation is involved in AML stemness via the activity of the enhancer of zeste homolog 2 (EZH2) subunit (89). EZH2 methylates H3K27, a modification involved in the repression of gene expression (90). During early disease development, EZH2 activity is necessary for epigenetic repression of differentiation, positively impacting AML survival and proliferation (91). However, a recent study showed that *EZH2* loss-of-function mutations led to increased chemotherapy resistance in AML cell lines. This is caused by the upregulation of EZH2 target genes involved in proliferation, apoptosis evasion, and membrane transport (92). This discrepancy largely occurs due to the context- and stage-dependent roles of the enzyme, as well as the utilization of different disease models. In particular, one study describes the importance of EZH2 in progenitor cells and the other in therapy-stressed cell lines with a different adaptation profile. In addition, different therapeutic modalities and model systems can cause an entirely distinct transcriptomic profile in the cells, which dictates the function of EZH2.

EZH2 activity is associated with other hematological cancers, such as MM, B-cell non-Hodgkin lymphoma, FL, CML, and natural killer/T-cell lymphoma (93–97). In B-cell lineage malignancies, EZH2 activity seems to be oncogenic, whereas enzymatic loss of function seems to be prevalent in myeloid disorders such as CML (98, 99).

In MM patients, *EZH2* is often overexpressed and correlates with a more aggressive disease and shorter overall survival, independent of treatment modality. This overexpression causes transcriptional repression of cell cycle regulatory genes, leading to increased malignant proliferation (90). In accordance with this, the pharmacological inhibition of EZH2 in MM cell lines and primary cells confers increased drug sensitivity, inhibits cell growth and can lead to a reactivation of certain tumor-suppressive regulatory genes (100, 101). The role of EZH2 in hematological cancers is both stage- and lineage-dependent. Increased H3K27 methylation drives stemness and proliferation in early AML, B-cell lymphomas and MM, while loss of function can promote resistance in CML and treated AML. Although FDA-approved EZH2-targeted therapy is already in use for FL (102), we are yet to elucidate under which conditions and in which diseases its inhibition will have a successful effect.

Another lysine methyltransferase, Suppressor of Variegation 3–9 Homolog 1 (SUV39H1), is responsible for the maintenance of constitutive pericentric heterochromatin. As a result, repetitive DNA sequences are transcriptionally repressed and regulated (103). Dysregulation of SUV39H1 disrupts the balance of H3K9 methylation, promoting the development of hematological malignancies (104).

The function of SUV39H1 has been investigated mostly in AML, where it exerts both tumor suppressive and oncogenic roles (104, 105). Specifically, *SUV39H1* expression was decreased in CD34^+^ AML cells compared to their healthy counterparts, resulting in an aberrant distribution of H3K9me3 (104). Deregulation of H3K9me3 in AML patients is well recognized and serves as a prognostic marker, when combined with clinical data (106). The repression of *SUV39H1* and the resulting reduction of H3K9me3 leads to reduced repression of oncogenes, such as *HOXB13*, *SIX1*, and *HOXA9*, whose downstream targets drive self-renewal and proliferation. Restoring the expression of *Suv39h1* halts disease progression and reduces the occurrence of leukemia stem cells in a murine model (104). The loss of *SUV39H1* seems to sustain immature stem cell persistence by disrupting oncogene repression, enabling leukemic self-renewal and therapy resistance. Conversely, the activity of SUV39H1 in AML cells led to promoter histone methylation of tumor suppressor genes such as *CDKN2B* and E-cadherin (*CDH1*). The inhibition of the SUV39H1 enzyme restored their transcription. However, this study specifically investigated MLL-AF9 AML (MLL is fused with ALL1-fused gene from chromosome 9, *AF9*), which likely has a distinct epigenetic landscape that influences SUV39H1 behavior (107). In addition, the differences between these two studies are major and relate to varying experimental models (murine vs. primary samples harboring specific mutations), gene/enzyme repression strategies and investigative methodologies. This highlights the challenges in defining the precise function of a protein in a disease, yet doing so remains essential for developing effective therapies.

A similar pattern of epigenetic dysregulation is observed in MM, where nuclear receptor binding SET domain protein 2 (NSD2) or multiple myeloma SET domain protein (MMSET), is a histone methyltransferase overexpressed in cells with the t (4, 14) translocation (108). This results in a global increase of H3K36me2 and drastic gene expression alterations and is associated with a poor prognosis (108, 109). Due to the role of MMSET in the DNA damage response, this enzyme is also involved in treatment resistance in MM cells (110). Knockout or knockdown of *MMSET* in t (4, 14) MM cell lines leads to apoptosis, cell cycle arrest, and reduced tumor growth *in vivo*, highlighting the importance of this enzyme in MM cell survival (111). In MM cells with increased MMSET expression, a nota ble increase in EZH2 recruitment was observed, further contributing to myeloma development and survival (112). This reveals a broader pattern of cooperative epigenetic regulation involved in myeloma malignancy.

Histone demethylases are studied less in hematological cancers. However, overexpression of lysine-specific demethylase 1 (*LSD1* or *KDM1A*) has been observed in AML cells (113). *LSD1* is considered an important mediator of the differentiation block of MLL, characterized by rearrangements in the *KMT2A* gene and is correlated with a poor clinical outcome. This enzyme removes methyl groups from H3K4 and acts as part of a transcription repression complex (114). Through its activity, LSD1 represses the transcription of genes involved in myeloid differentiation, contributing to AML stemness.

In line with that, the inhibition of LSD1 leads to the differentiation of primary murine and human MLL cells, without affecting healthy hematopoietic progenitor cells (115). In addition, knockdown of *LSD1* in AML cell lines led to a reduction in their clonogenic potential and proliferation. *In vivo LSD1* knockdown also induced an upregulation of integrin alpha M (CD11b, *ITGAM*) and CD86 in AML, which are recognized myeloid differentiation markers (114). However, several studies showed the function of CD11b and CD86 in tumor progression (114, 116–118). Based on current knowledge, the role of CD11b is stage dependent – high baseline expression of CD11b indicates an aggressive leukemia phenotype, whereas therapy-increased CD11b is considered a good response, as it is associated with a differentiation block exit. Studies correlating the baseline expression of CD11b with a poor outcome in AML patients are mostly cohort studies and meta-analyses which do not discuss the molecular mechanisms behind this occurrence.

Likewise, *LSD1* is overexpressed in other myeloid diseases, such as myeloproliferative neoplasms, CML, and MDS (119). The relevance of *LSD1* in myeloid diseases suggests a shared dependency of epigenetic repression to differentiation and lineage fidelity.

In ALL, targeting LSD1 in a 3D culture setting increased the invasive capacity in cell lines, an effect opposite to that observed *in vivo* studies. In fact, LSD1 inhibition seems to have decreased the colonization potential of ALL cells *in vivo*, with impaired chemotaxis and migration (120). These findings emphasize the importance of contextual cues and the investigation of tumor cells in their native environment. Moreover, although useful, cell lines do not necessarily represent the original cancer ideally, and certain studies may therefore yield contrasting results.

Selective oncogenic pressure leads to an overexpression of histone methyltransferases that repress tumor suppressors and histone demethylases whose activity promotes the transcription of genes involved in self-renewal and proliferation. The activity of these enzymes is dictated by other proteins in the cells, which recruit them to specific sites. This explains the observed effects in cancer cells, but not in their healthy counterparts that have a different proteomic milieu. The transcriptional and proteomic profiles of these cells have not yet been investigated in such a systematic manner, but only correlated to genes involved in standardized cancer pathways – proliferation, apoptosis, self-renewal, etc. Therefore, gaps remain regarding a unified vision of how these processes mutually interact, emphasizing the need for integrative *in vivo* and clinical studies, combining them with current knowledge.

### 3.2 Histone acetylation in hematological malignancies

Histone acetylation is considered to activate transcription due to the loosening of chromatin structure, which makes DNA accessible to transcriptional regulators. Histone acetylation is mediated by two families of enzymes: histone acetyltransferases (HATs), which promote transcription, and histone deacetylases (HDACs), which repress transcription. The equilibrium between the activity of HATs and HDACs maintains normal hematopoietic differentiation, and its disruption contributes to hematopoietic malignancies (121). These enzymes are nowadays more often referred to as KATs and KDACs, to emphasize the fact they also (de)acetylize lysine residues on other proteins as well (122).

In hematological cancers, HATs are mutationally inactivated. For instance, a histone acetyltransferase gene, CREB-binding protein (*CREBBP*, or *KAT3A* encoding CBP/p300), was found to be mutated in 20% of relapsed ALL patients. These mutations were either present at diagnosis in certain subpopulations or acquired during relapse, indicating a correlation between HAT dysfunction and disease persistance. Observed *CREBBP* mutations affected the acetyltransferase domain, resulting in decreased H3K18 or H3K27 acetylation. This led to a dysregulated acetylation of promoters or enhancers of genes responsive to glucocorticoids, involved in the response to corticosteroid treatments, possibly rendering these cells resistant to the therapy (123).

Besides ALL, *CREBBP* mutations were found in other lymphoid malignancies, such as diffuse large B-cell lymphoma (DLBCL) and FL patients (124). This suggests a shared vulnerability of lymphoid-lineage cancers to dysregulated histone acetylation.

However, in myeloid malignancies, such as AML, the activity of CBR/p300 is linked to increased self-renewal, which can be reduced when an enzymatic inhibitor is applied (125). This dual role of CBR/p300 across different hematological malignancies may occur due to different lineage origins and differentiation stages. Either way, both CBR/p300 inhibition and restoration of activity can be beneficial, based on disease modality or mutational context, complicating future therapeutic strategies.

Overexpression of HDACs, rather than specific mutations, was detected in hematological malignancies. For example, *HDAC1* was overexpressed in AML cells, which was linked to an oncogenic transcription regulation. HDAC1 negatively correlates with the Kruppel-like factor 4 (Klf4) tumor suppressor by competing for its promoter and inhibiting its expression. The reduction of Klf4 in turn reduces the expression cell cycle regulators, cyclin-dependent kinase inhibitors p21 (*CDKN1A*) and p27 (*CDKN1B*), and promotes proliferation. In addition, lower *HDAC1* expression in AML patients was associated with longer overall and disease-free survival (126).

In addition to aberrant expression levels, HDACs are often recruited by pathogenic fusion proteins in acute leukemias. In AML and acute promyelocytic leukemia (APL) pathogenic fusion proteins are frequent and occur due to chromosomal translocations. The dysregulated engagement of HDACs leads to a suppression of tumor suppressor genes and, ultimately, to differentiation arrest and leukemia development (127, 128). While this general principle was established long ago, the specific tumor repressors targets and their contribution to pathogenesis are under-investigated.

HDAC-mediated transcriptional repression has been recognized in other hematological malignancies as well. Upregulation of HDACs is also observed in B-CLL cells, compared to healthy controls. These enzymes seem to play a complex role in CLL development, acting as both activators and repressors of transcription, depending on chromatin organization. Specifically, when HDAC1 is recruited to super-enhancers, it functions as a transcriptional activator of genes involved in CLL survival, such as B-cell lymphoma 2 (*BCL2*). Conversely, in regions without super-enhancer marks, HDAC1 represses transcription, particularly of tumor suppressor genes (129). This reveals a very high level of oncogene/tumor suppressor expression regulation, dependent on chromatin organization, since the proteins involved in guiding *HDAC1* through other epigenetic marks also function in a protumorigenic manner. This study once again emphasizes the complexity of investigating the involvement of specific epigenetic regulators in one disease entity and underscores the necessity for a more complex, contextual, proteomic analysis.

In CML, *HDAC* overexpression is correlated with a drug-resistant population of cells and an increased expression of multipotent stemness genes such as SRY (sex-determining region Y)-box 2 (*SOX2*), homeobox transcription factor NANOG (*NANOG*), and octamer-binding transcription factor 4 (*OCT4*), which leads to disease persistence and treatment evasion (130).

In lymphomas, class I HDACs are broadly expressed and facilitate immune evasion and tumor suppressor gene silencing (131). Similarly to lymphomas, class I HDACs are dysregulated in MM, which is correlated with a poor prognosis (132).

Given the broadly established role of HDACs in lymphoid and myeloid malignancies, HDAC inhibitors such as vorinostat, belinostat, and romidepsin have already been approved for the treatment of hematological cancers as combinational therapies, but have failed to yield significant results (133).

Dysregulation of histone acetylation, whether through HAT mutations or HDAC overexpression plays an important role in hematological malignancies by regulating the expression of oncogenes and tumor suppressors. The effects of these enzymes are context dependent and vary by disease subtype.

## 4 Chromatin remodeling complexes in hematological malignancies

**Figure 3:**
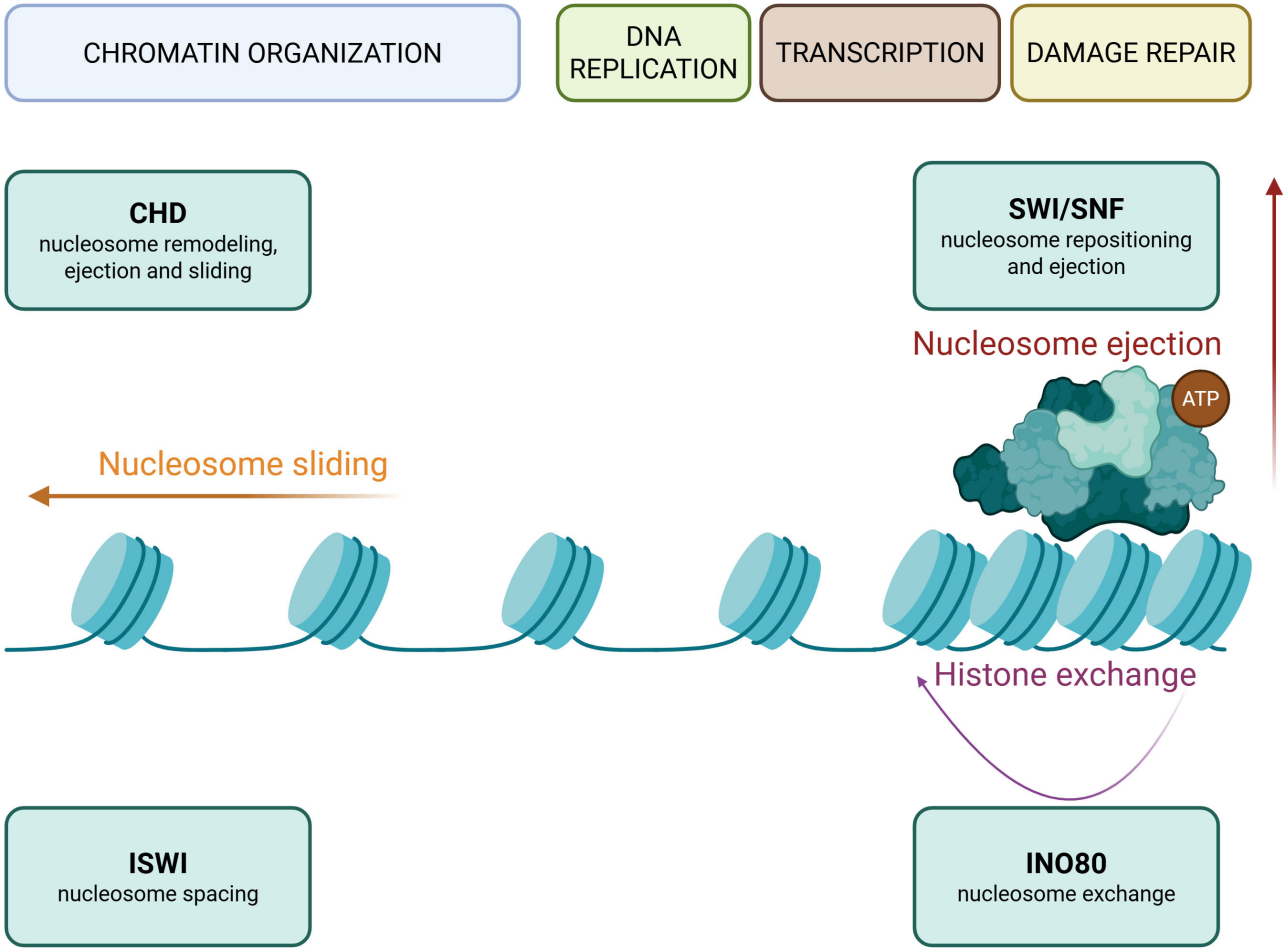
Chromatin remodeling complexes and their functions. Schematic representation of chromatin remodeling complexes SWI/SNF, ISWI, CHD, and INO80 that regulate nucleosome organization and chromatin accessibility. Chromatin remodeling proteins utilize ATP hydrolysis to organize the chromatin through nucleosome repositioning, ejection or sliding or nucleosome/histone exchange. The CHD family mediates nucleosome sliding and eviction, while ISWI complexes primarily regulate nucleosome spacing. SWI/SNF complexes are involved in nucleosome repositioning and ejection, opening chromatin. INO80 proteins facilitate histone exchange, including the replacement of canonical histones with histone variants. The activity of chromatin remodeling complexes regulates key DNA-dependent processes, including replication, transcription and DNA damage repair. Image created in Biorender.

Chromatin remodeling refers to dynamic changes in chromatin structure, which interact with DNA methylation and histone modifications. It dictates the spatial organization of nucleosomes and, thus, the transcriptional availability of DNA. There are four major families of chromatin remodeling complexes, comprised of ATP-dependent enzymes: Switch/Sucrose Non-Fermenta ble (SWI/SNF), Imitation Switch (ISWI), Chromodomain-Helicase-DNA-Binding (CHD), and Inositol-Requiring 80 (INO80). Chromatin remodeling complexes are responsible for nucleosome repositioning, ejection, and restructuring which changes the accessibility of DNA for transcription (**Figure 3**) (134).

### 4.1 SWI/SNF chromatin remodeling complexes in hematological malignancies

SWI/SNF complexes are comprised of numerous subunits and are involved in nucleosome repositioning and ejection, which opens chromatin and exposes promoter regions. Once recruited to DNA, specific histone modifications or transcription factors, the ATPase subunits utilize ATP hydrolysis energy to slide nucleosomes along DNA or eject them. This alters the chromatin structure, which either increases or restricts DNA accessibility. In this manner, SWI/SNF proteins modulate the ability of transcription factors and the RNA polymerase to interact with regulatory elements. SWI/SNF complexes regulate DNA replication, transcription, and genomic stability, guiding normal cellular development and differentiation. When these processes are dysregulated, they can contribute to the emergence of cancer. In hematology, SWI/SNF complexes function as tumor suppressors in lymphoid cancers via loss-of-function mutations, while acting as oncogenes in myeloid malignancies through gain-of-function mutations or overexpression (135).

In myeloid diseases, such as AML, mutations in SWI/SNF enzymes are rare, but their activity is correlated with cellular differentiation and patient survival. The SWI/SNF subunit SWI/SNF Related BAF Chromatin Remodeling Complex Subunit D1 (SMARCD1) is highly expressed in CD34^+^ hematopoietic cells, which decreases during regular myeloid differentiation. Increased SMARCD1 expression in AML patients suppresses myeloid differentiation via repression of transcription regulators of myeloid differentiation, such as CD11b, CD14 (monocyte/macrophage marker), S100A8/A9 (S100 calcium-binding proteins), and CCL3/4/7/8 (chemokine C-C-motif ligands). This repression is mediated by decreased histone methylation on H3K4 and increased histone methylation on H3K27. In this manner, *SMARCD1* is involved in the differentiation block of AML, and its overexpression correlates with a stem cell signature and poor prognosis, promoting leukemogenesis and disease maintenance (136).

In contrast, in lymphoid malignancies, SWI/SNF complex proteins act as tumor suppressors via loss of function mutations. For instance, in DLBCL and FL, nonsense or frameshift alterations occur in the AT-rich interactive domain-containing protein 1A (ARID1A) subunit recurrently, truncating the protein (135, 137). In B cells, ARID1A chromatin remodeling activity assists the binding of transcription factors (such as nuclear factor kappa-light-chain-enhancer of activated B cells, NF-κB) to genes involved in B-cell germinal center differentiation and activation. Loss of *ARID1A* impairs B-cell maturation, influencing both patient immunity and lymphoma development (138).

Furthermore, in FL, nearly all patients carry mutations in chromatin remodeling genes, with *ARID1A* and *SMARCA4* being the most common. It remains unclear why FL is particularly vulnerable to dysregulated chromatin remodeling. In fact, other epigenetic regulators, such as *KMT2B*, *CREBBP* and *EZH2* are frequently mutated in this disease as well. It is speculated that FL’s dependence on epigenetic mutations stems from their germinal center (GC) B-cell origin, characterized by heavy proliferation and high mutational burden, with an increased tolerance for DNA damage. Thus, the mutations occurring in epigenetic regulators are tolerated as and can cumulatively give rise to malignant cells (139).

*SMARCA4* mutations are very common in B-cell lymphomas as well. In germinal GC-derived Burkitt lymphoma, *SMARCA4* mutations are found in 30% of patients. *SMARCA4* is involved in regular GC B cell development. Monoallelic loss of *SMARCA4* decreases the activity of transcription factors needed for GC exit, therefore disrupting normal B-cell development, which can ultimately lead to carcinogenesis. Recurrent mutations in SWI/SNF subunits such as *ARID1B/2*, *SMARCA2* an*d SMARCA4* are observed in hematological cancers as well, indicating the important role these proteins have in oncogenesis. In addition, mutations in SWI/SNF genes were discovered in Burkitt lymphoma, Hodgkin lymphoma and other B, T and NK-cell lymphomas (135). These mutations are not equally distributed, but highlight a vulnerability of lymphoid cells to SWI/SNF dysregulation. However, it is still unclear whether these mutations initiate oncogenesis or emerge as secondary events, supporting neoplastic cells.

In MM, mutations in the BAF Chromatin Remodeling Complex Subunit 7A (*BCL7A*) SWI/SNF subunit gene were identified in non-coding regions in about 60% of patients. *BCL7A* is downregulated in MM cells compared to healthy plasma cells and is considered to have a tumor suppressive role, since loss of *BCL7A* led to increased MM growth and viability. BCL7A protein interacts with the Interferon Regulatory Factor 4 (IRF4) transcription factor to limit its function. Loss of *BCL7A* leads to the transcription of IRF4-targeted genes, which drives MM cell growth (140). Therefore, the loss (or lack) of *BCL7A* leads to IRF4 activation, promoting genes involved in MM proliferation. The specific target genes of IRF4 in MM are unknown. This study highlights the importance of mutations in non-coding regions, raising the question of their potential relevance in other epigenetic regulators, which might have been overlooked in whole-exome sequencing approaches thus far.

Mutations in SWI/SNF subunits are rare in leukemias, except for T-ALL, where *BCL11B* subunit mutations are speculated to be a driver of the disease and occurs fairly frequently – in about 10% of patients. *BCL11B* mutations are linked to all major subtypes of T-ALL, most commonly as monoallelic deletions or missense mutations. It is speculated that these mutations in T-ALL correlate with mammalian target of rapamycin (mTOR) signaling activation, generally involved in proliferation and survival, though exact mechanistic links are not described (141).

In summary, SWI/SNF dysregulation in hematological malignancies is involved in transcriptional changes, resulting in impaired differentiation and the maintenance of stemness in cancer cells. Despite the involvement of distinct subunits across different malignancies, these effects arise through diverse cellular mechanisms.

### 4.2 ISWI chromatin remodeling complexes in hematological malignancies

Subunits of the ISWI chromatin remodeling complex bind to nucleosomes and slide them along DNA, providing adequate nucleosome spacing, which limits chromatin accessibility and transcription (134, 142). ISWI complexes are comprised of either the SMARCA5 or SMARCA1 catalytic subunits, which translocate DNA through ATP hydrolysis, without disassembling the nucleosome (143, 144). This translocation occurs in a repetitive manner, through cycles of sliding and pausing, resulting in regularly spaced nucleosomes, which organizes the chromatin structure (145). Defects in ISWI function contribute to genomic instability and have been associated with cancer (146). In addition, the activity of ISWI complexes is required for proper hematopoietic differentiation and therefore essential for normal blood cell development (125).

In myeloid malignancies, altered ISWI activity is correlated with impaired differentiation and disease persistence. For example, *SMARCA5*, or Sucrose Non-Fermenting 2 Homolog (*SNF2H*), was overexpressed in AML CD34^+^ bone marrow cells, compared with their healthy counterparts, and was likely to contribute to differentiation dysregulation. When patients were in remission, the expression of *SMARC5* was reduced, indicating a correlation between the enzyme with disease activity (147). The overexpression of *SMARCA5* leads to an epigenetic repression in the myeloid differentiation regulator PU.1 in both AML cell lines and primary samples (148). Additionally, the deletion of *SMARCA5* in AML cell lines led to cell cycle arrest, indicating its role in the maintenance of this cancer (149). Given that ISWI complexes are involved in the regulation of DNA repair, it is likely that the proliferation halt in these cells occurs due to the accumulation of DNA damage (144). Since ISWI complexes are responsible for genome integrity, their activity is crucial for cellular survival and proliferation.

In lymphoid malignancies, ISWI subunits exhibit oncogenic activity as well. For instance, Bromodomain Adjacent To Zinc Finger Domain 2A (*BAZ2A*), was overexpressed in CLL patients compared to respective healthy controls, due to the downregulation of tumor suppressor miRNAs (150). Even though the ISWI complex is essential for cellular integrity, the overexpression of *SMARCA5* in AML patients and *BAZ2A* in CLL patients indicates their oncogenic role, as it might influence the well/calibrated proteomic balance in the cell and lead to dysregulation in genome organization.

The overexpression of ISWI subunits is found in both AML and CLL, despite their myeloid and lymphoid origins, suggesting a shared oncogenic mechanism. Although these studies identified ISWI protein involvement in several hematological cancers, mechanistic input or clinical relevance are not precisely described. There aren’t many studies characterizing the function of the ISWI complex in other hematological malignancies. However, its role in hematopoiesis and its oncogenic function suggests relevance in these cancers and warrants further investigation.

### 4.3 CHD chromatin remodeling complexes in hematological malignancies

The CHD family of proteins organizes histone octamers into stable nucleosomes upon replication. They are involved in nucleosome ejection or sliding, as well as histone deacetylation. The interaction of CHD proteins with modified histone tails, DNA, or transcription factors leads to the activation of their ATPase activity, which in turn slides or repositions nucleosomes. CHD proteins use ATP hydrolysis to twist the DNA around the histones, which changes DNA accessibility and transcription factor binding, regulating transcription. It is uncertain whether CHD proteins directly interact with other epigenetic proteins, such as DNMTs or TETs, but they do depend greatly on their activity, as they are recruited through the recognition of specific histone modifications (151).

Their role in hematological cancers has not been investigated thoroughly. Recent studies show that certain members of this family, such as *CHD4*, *CHD7*, and *CHD9* are overexpressed in AML patients, suggesting their tumor promoting roles in myeloid lineage cells (152). *CHD4* regulates the expression of the *MYC* oncogene in AML, promoting cell cycle progression Additionally, shRNA knockdown of *CHD4* in AML cell lines opens the chromatin, increasing its susceptibility to double-stranded breaks when genotoxic treatments are applied (153). The correlation between the expression of CHD proteins and AML patient clinical parameters suggests their relevance in this disease, but the data lacks conviction (152).

In contrast, *CHD2* gene was mutated in CLL patients, suggesting its tumor suppressive role. *CHD2* mutations result in reduced association with chromatin, leading to transcriptional deregulation of genes involved in DNA damage repair and lymphocyte differentiation. These mutations often coincide with mutations in immunoglobulin heavy chain variable region genes, which are correlated to a better prognosis and usually indicate indolent disease. This raises the question of the clinical relevance of CHD mutations in CLL, which remains to be elucidated (154).

In leukemias, *CHD5* seems to have a tumor suppressive role. *CHD5* is silenced via promoter hypermethylation in CML, suggesting a cooperative link between different epigenetic mechanisms to promote leukemogenesis. In CLL cells, *CHD5* restoration led to cell cycle arrest and promoted apoptosis due to the upregulation of the p21 cell cycle regulator. *In vivo*, *CHD5* overexpression reduced cancer cell growth (155).

Limited data suggest that certain CHD proteins may function as oncogenes in plasma cell malignancies. One *in vitro* study was conducted regarding the involvement of *CHD1L* in MM. It was observed that *CHD1L* has an anti-apoptotic function in MM cell lines and that its overexpression led to increased cell adhesion/mediated drug resistance. This suggests that *CHD1L* might contribute to drug resistance in MM. However, this study was only conducted on cell lines, and more investigation is warranted to confirm these findings (156).

### 4.4 INO80 chromatin remodeling complexes in hematological malignancies

INO80 chromatin remodeling complex is involved in histone sliding and ejection, as well as histone exchange. By repositioning or evicting nucleosomes, INO80 proteins modulate chromatin organization, which influences replication, transcription and DNA repair. INO80 exchanges the histone variant H2A.Z with canonical H2A in nucleosomes, which changes promoter and enhancer accessibility (157). Specifically, H2A.Z-containing nucleosomes are less stable, resulting in a more plastic chromatin structure. This is associated with transcriptional activation and a high responsiveness to molecular signaling (158). Several studies have observed increased H2A.Z chromatin presence in various cancers, contributing to tumor growth, drug response, and metastasis (159–161).

The evidence about INO80 involvement in hematological malignancies is scarce and their role in these cancers remains speculative. Transcriptomic data identified mutations of INO80 in DLBCL, along with other genes whose protein products are linked to chromatin maintenance and regulation, such as *EZH2*, *CREBBP*, *TET2*, *DNMT3A* and others (162). It seems that epigenetic dysregulation is a hallmark of DLBCL. No mechanistic or clinically relevant data were acquired regarding INO80 proteins in this cancer. Given the vulnerability of hematological malignancies to epigenetic dysregulation, INO80 is likely implicated in their biology, although this remains uninvestigated.

Overall, chromatin remodeling complexes regulate gene accessibility and expression. Dysregulation of SWI/SNF, ISWI, CHD, and INO80 complexes leads to impaired differentiation, increased stemness, and the activation of oncogenic transcriptional programs in hematological malignancies. While SWI/SNF complexes often harbor mutations in lymphoid malignancies and are overactivated in myeloid malignancies, other complexes exhibit context-dependent roles. Given their ubiquitous expression, therapeutic targeting seems unlikely. Nonetheless, a systematic investigation into their interaction with other epigenetic regulators is essential in understanding cancer cell plasticity and adaptability. In this manner, we would identify additional vulnerabilities that could be therapeutically exploited indirectly.

## 5 Interplay between epigenetic mechanisms in hematological cancers

**Figure 4:**
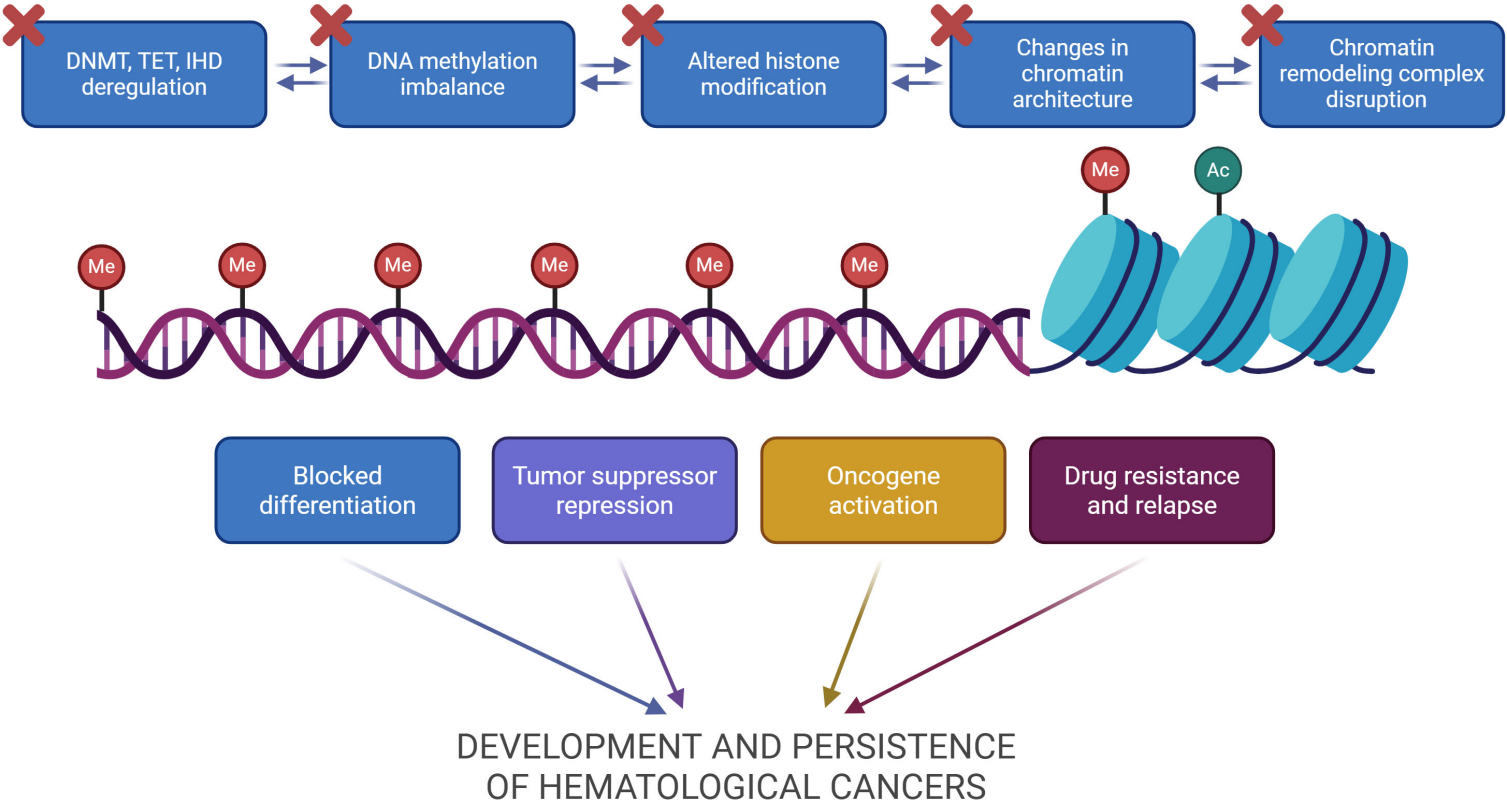
Integrated model of epigenetic dysregulation in hematological cancers. Changes in DNA methylation enzymes, structure of chromatin, histone modification enzymes, and chromatin remodeling complexes act synergistically to contribute to the development of hematological cancers. These alterations often reinforce one another through positive feedback loops, resulting in widespread epigenetic dysregulation. For instance, dysregulation of DNA methylation proteins leads to altered DNA methylation patterns. This influences the recruitment of histone modifiers, changes chromatin architecture and chromatin organization complex activity. The combined effects of these alterations contribute to key oncogenic processes, such as blocked differentiation, tumor suppressor repression, oncogene activation, and drug resistance, which together promote oncogenesis and persistence of hematological cancers. Red crosses indicate dysregulation. Image created in Biorender.

Epigenetic mechanisms act as a complex network of proteins that regulate gene expression. Coordinated crosstalk between DNA methylation, histone modifications and chromatin organizing complexes defines all cellular functions. Their dysregulation can promote tumor development and growth (**Figure 4**). Although many studies have investigated numerous epigenetic regulators in hematological cancers, it is still unclear how exactly their interaction functions to orchestrate neoplastic transformation.

Distinct DNA methylation patterns recruit DNMTs in a specific, context-dependent manner. In hematological cancers, *DNMT3A* mutations are common, which cause genome-wide hypomethylation, removing these marks and disturbing regular DNA methylation patterns. This modulates the function of histone methyltransferases, whose activity is also dependent on DNA methylation (163, 164). For instance, in AML, mutations in DNA methylation proteins such as DNMT3A, IDH1/2, or TET2 enzymes often co-occur and cooperate to drive leukemogenesis (41). *IDH2* mutations are also linked to histone hypomethylation in AML, which influences the activity of histone methyltransferases (163). In ALL, the histone methyltransferase *KDM2B* is upregulated, acting cooperatively with HATs to induce oncogene expression (165). Increased *MMSET* expression in MM cells is associated with enhanced EZH2 recruitment, supporting myeloma progression and survival (112). Data about interactions of epigenetic modulators converging on specific genes is scarce, limiting our understanding of how these layers of regulation interact to control oncogenic and tumor-suppressive programs. Taken together, the deregulation of DNA methylation, histone modification, and chromatin remodeling complexes are observed in hematological malignancies. We are yet to determine how exactly all of them interact with each other to obtain a clear picture of disease pathogenesis and maintenance. The changes in DNA methylation influence histone modification and vice versa. This, in turn, changes the chromatin structure and DNA accessibility which further dictates the activity of these enzymes and chromatin remodeling complexes and their mutual interactions.

To systematically present epigenetic changes in hematological cancers, their summary was provided in **Table 1**.

**Table 1:** Epigenetic changes in hematological cancers and their functional consequences.

Disease	AML	ALL (B/T)	MDS
DNA (de)methylation enzymes	High frequency of mutations in *DNMT3A, TET2, IDH1/2* genes (21, 36, 54). *TET2* and *IDH1/2* mutations are mutually exclusive (57). Indication of *DNMT3B* tumor suppressive role (12, 33).	Less frequent *DNMT/TET/IDH* mutations than AML. *DNMT3A* suspected of oncogenic activity (34).	High incidence of *DNMT3A*, *TET2*, and *IDH1/2* mutations (21, 44, 49).
DNA methylation pattern	Global hypomethylation and promoter hypermethylation of tumor suppressors, used as a prognostic marker (65).	Hypomethylation of tumor suppressor genes (75).	The progression of MDS to AML is correlated with DNA methylation of tumor suppressor genes (63).
Histone modifications	*KMT2A* mutations which increase upon treatment (88). Dual *EHZ2* role, dependent on disease stage (89). Dual *SUV39H1* role (104, 107). Increased *LSD1*, *CREBBP*, *HDAC1* overexpression (114, 123, 126)	*KMT2A* translocation leads to aberrant H3K4 methylation and can be used as a prognostic marker (83). LSD1 suggested to be involved in cancer invasiveness (120). High frequency of mutations in *CREBBP* relapsed patients (123).	Overexpression of *LSD1* (119).
Chromatin remodeling complexes	High expression of *SMARCD1* and *SMARCA5* (136, 149). *CHD4*, *CHD7*, and *CHD9* are overexpressed (152).	*BCL11B* mutations considered to be the driver of disease when they occur (141).	Less frequent than AML, suggesting a developmental role of these lesions.
Functional consequences	Blocked differentiation and persistence of drug-resistant clones (24).	Pathological progenitor self-renewal and impaired differentiation (87)	Increased self-renewal and survival of drug-resistant clones (166).
Disease	CLL	Multiple myeloma (MM)	Lymphomas (B/T, FL, DLBCL, AITL)
DNA (de)methylation enzymes	No distinct DNMT/TET/IDH mutations​ have been identified.	*DNMT1* overexpression (29); *DNMT3A* loss-of function mutations (21, 25, 26).	*DNMT*/*TET* mutations are uncommon in most B-cell lymphomas. In AITL *TET2* mutations occur with a high frequency, often together with *DNMT3A* (61).
DNA methylation pattern	Methylation patterns do not correlate with therapy response (76).	Global hypomethylation with focal hypermethylation of tumor suppressors and differentiation regulators; epigenetic remodeling drives MGUS to MM (26, 68).	DNA methylation as a prognostic marker in B-cell lymphoma (67).
Histone modifications	Upregulated *HDAC1* expression, with a complex, chromatin-dependent role (129).	*EHZ2* overexpression (90). Overexpression of MMSET in patients with t (4, 14) translocations (110).	Oncogenic increase of EHZ2 activity across lymphomas (93–97). *CREBBP* mutations in DLBCL and FL (124).
Chromatin remodeling complexes	*BAZ2A* overexpression (150) and mutations in CHD2 (154).	Very high frequency of *BCL7A* mutations (140).	*ARID1A* nonsense or frameshift mutations common in DLBCL and FL (135, 137). *ARID1B/2*, *SMARCA2* and *SMARCA4* occur high frequency of mutations in lymphomas (135). *INO80* mutations coinciding with *CREBBP*, *TET2*, and *DNMT3A* (162).
Functional consequences	DNA damage repair and lymphocyte differentiation gene expression dysregulation (154).	Increased self-renewal, inhibition of apoptosis, repression of tumor suppressors and B-cell enhancers, oncogene overactivation (29, 69, 70).	Disrupted normal B-cell development, inability to exit the GC (FL and DLBCL) (135).

The overarching epigenetic patterns in hematological malignancies involve suppression of cell differentiation, leading to more immature, less differentiated phenotypes. Furthermore, epigenetic regulation and chromatin architecture repress tumor suppressors and promote oncogene expression. This promotes cancer cell survival, resulting in more drug-resistant clones with the ability to relapse. Given the dynamic nature of epigenetic marks, this occurs due to cumulative selection of cells with adaptive behavior during cancer development and subsequent therapeutic pressure.

## 6 Clinical translation and therapeutic implications of epigenetic alterations

Most epigenetic therapeutics approved thus far are used in hematological malignancies, due to the relevance of epigenetic dysregulation in these cancers. Currently approved FDA therapies involve DNMT, HDAC, IDH, and EZH2 inhibitors (167).

DNMT inhibitors azacitidine and decitabine, which target DNMT1 primarily, were among the first epigenetic drugs approved and are currently used in the treatment of AML, MDS and CML. These drugs induce hypomethylation in cancer cells, which can lead to the expression of silenced tumor suppressor genes. However, hypomethylation caused by these inhibitors does not correlate with clinical outcome, and is often transient, resulting in attenuated therapeutic efficacy (168, 169). Rapid restoration of methylation patterns, upregulation o*f DNMT1* and *DNMT3B* and metabolic drug manipulation can all contribute to resistance to DNMT inhibitors (170). Even though DNMT enzymes are upregulated in many hematological cancers and used as monotherapy in some cases, their sole inhibition does not eliminate cancer cells long-term. The question arises whether the problem is their activity per se, or rather a dysregulated distribution of DNA methylation that needs to be restored.

HDAC inhibitors vorinostat, romidepsin, belinostat, and panabinostat increase histone acetylation which opens chromatin and reactivates silenced tumor suppressor genes, which can lead to cell cycle arrest and apoptosis (171). Vorinostat is approved for relapsed/refractory CTCL; romidepsin for CTCL and peripheral T−cell lymphoma (PTCL); belinostat for relapsed/refractory PTCL; and panobinostat, in combination with bortezomib and dexamethasone, for relapsed/refractory MM (167). HDAC inhibitors used as monotherapy have failed to yield significant results, since patients rarely achieve complete remission or long-term disease-free survival (171, 172). The insufficient efficacy of these therapeutics could be attributed to various factors. HDACs deacetylate other proteins as well, involved in apoptosis, DNA repair, and survival. The inhibition of HDACs increases acetylation on other proteins, which can result in their (increased) activation. This can provide an additional advantage to cancer cells by promoting broader resistance phenotypes. In addition, cancer cells treated with HDAC inhibitors can upregulate anti-apoptotic proteins, increase DNA damage response, or employ antioxidant defense pathways (173).

FDA-approved IDH inhibitors are enasidenib, ivosidenib and olutasidenib, used for the treatment of AML patients with recognized IDH mutations. These drugs specifically target mutated IDH1 or IDH2 proteins. They lovers 2-HG levels in the cells, restoring TET function and releasing the differentiation block. These therapies represent a good example of precision medicine, where only mutated isoforms are targeted, minimizing the therapeutic influence on healthy cells. Since their efficacy as monotherapies has proven insufficient, they are planned to be integrated into combination therapies (174). Given that these therapeutics have been recently approved, not much is known about the mechanisms behind drug response. However, in AML treated with IDH inhibitors, leukemia cells are sometimes found to switch between IDH1 or IDH2 mutations at relapse, evading targeted therapy (175).

EZH2 inhibitor tazemetostat is used in the treatment of FL. The inhibition of EZH2 leads to reduced H3K27 methylation and derepression of differentiation-related and tumor suppressor genes. Tazemetostat inhibits both mutant and wild−type EZH2, leading to cell cycle arrest, apoptosis, and inhibited tumor growth in lymphoma cells. Thus far, tazemetostat seems to be well-tolerated and effective, with a potential to be used in combination therapies for improved clinical outcomes (102).

Other epigenetic drugs are currently under investigation as well. For example, inhibitors of the LSD1 histone demethylase are under investigation for the treatment of AML. LSD1 inhibitors promote differentiation in AML cells by increasing H3K4 methylation. They often only induce partial myeloid differentiation, and their activity can be compensated by other histone demethylases, which is why they exhibit limited treatment success as monotherapy. Therefore, combination therapies are under investigation, including strategies targeting signaling pathways involved in the maintenance of stemness (176). Development of novel epigenetic inhibitors is ongoing, generating next-generation drugs, with enhanced efficacy or targeting new epigenetic proteins altogether. Taken together, there is considerable potential in targeting epigenetic regulators, which clearly contribute to hematological cancers. To do so efficiently, a deeper understanding of epigenetic modalities is required—in both disease- and stage- specific context and their reciprocal interactions. It is likely that epigenetic drugs will not be effective as monotherapy but will need to be used in combination with other therapies to achieve meaningful clinical outcomes.

In addition, epigenetic biomarkers have emerged as powerful tools in the diagnostics, prognosis, and treatment of hematological malignancies. DNA methylation patterns define epitypes and methylation classifiers, which provide improved prognostic prediction. For instance, specific CpG island methylation signatures in AML, lymphoma, and MM have been shown to correlate with treatment response and survival outcomes (64, 66, 67, 74, 75). Integrating epitypes into clinical workflows is a good example of combining epigenetic insights into clinical practice and therapeutic strategies.

The future therapeutic use of epigenetic knowledge should integrate the development of drugs that precisely target relevant epigenetic regulators, together with the use of predictive models for patient stratification and precision medicine. The combination of epigenetic drugs with other therapeutical modalities may enhance efficacy and overcome resistance. Advances in patient-specific epigenetic profiling will enable personalized treatment strategies, improving outcomes while minimizing toxicity.

## 7 Conclusion

DNA methylation, histone modifications and chromatin remodeling are involved in the development and maintenance of hematological malignancies. Epigenetic gene expression regulation facilitates swift responses to alternating cellular signals, enabling a quick adaptation of the cell to changing environmental cues. This plasticity is exploited extensively by cancer cells contributing to treatment evasion and clonal evolution and ultimately, drug resistance and relapse. Therefore, future frameworks should integrate epigenetic regulation into cancer research, investigated with multi-omic approaches, AI predictive models, and chromatin conformation analysis to uncover the drivers of hematological malignancies and therapeutic resistance, offering significant potential for new discoveries.

Given the role epigenetic regulators clearly play in hematological malignancies, numerous epigenetic inhibitors are used for their treatment. Clinically approved epigenetic inhibitors are used in the treatment of hematological cancers, often in combination with other therapies, but their efficacy remains limited. In addition, it can be particularly challenging to target epigenetic regulators, due to their ubiquitous function. A more selective approach, such as targeting specifically mutated enzymes, could provide more favorable outcomes in patients in the future.

Even though epigenetic inhibitors are widely used in the treatment of hematological cancers, they have largely failed to achieve desired clinical outcomes. Recent studies of hematological cancers focus on immunotherapies and DNA mutational events, while overlooking the epigenetic context. A comprehensive understanding of these dynamic regulatory changes, their interactions, and integration into novel treatment approaches, is necessary to further improve treatment. Most studies investigate one particular epigenetic mark, due to obvious experimental limitations. However, it would be of significant value to establish libraries of specific histone code patterns, chromatin accessibility and DNA spatial organization, and to map their relevance in cancer progression. Future approaches should focus on providing a more integrative and systematic investigation in this field. For example, AI-based predictive frameworks could correlate chromatin conformation states with transcriptional outputs. This could predict how combinations of histone modifications shape enhancer–promoter interactions and identify epigenetic determinants of immunotherapy resistance. In addition, predictive AI models might reveal connections between epigenetic patterns and chromatin conformation states with proteomic and transcriptomic outputs. This can uncover epigenetic determinants and provide insights into specific cancer behaviors. In addition, data acquired from multi-omics approaches, such as ATAC-seq, ChIP-seq and single cell RNA-seq could help identify novel vulnerabilities, (epigenetic) cell heterogeneity and microenvironmental dependencies.

The extensive amount of data already collected has enormous potential to yield more definitive conclusions when summarized and put into a biological context by computational methods. Finally, the integration of discovered epigenetic markers could be utilized in precision medicine, by developing patient-specific profiles which could predict and improve treatment responses.

## References

[1] Lachmann M Libby E . Epigenetic inheritance systems contribute to the evolution of a germline. Philos Trans R Soc Lond B Biol Sci. (2016) 371:20150445. doi: 10.1098/rstb.2015.0445, PMID: 27431523 PMC4958939

[2] Jaenisch R Bird A . Epigenetic regulation of gene expression: how the genome integrates intrinsic and environmental signals. Nat Gen. (2003) 33:245–54. doi: 10.1038/ng1089, PMID: 12610534

[3] Holoch D Moazed D . RNA-mediated epigenetic regulation of gene expression. Nat Rev Genet. (2015) 16:71–84. doi: 10.1038/nrg3863, PMID: 25554358 PMC4376354

[4] Bachireddy P Burkhardt UE Rajasagi M Wu CJ . Haematological Malignancies: at the forefront of immunotherapeutic innovation. Nat Rev Cancer. (2015) 15:201–15. doi: 10.1038/nrc3907, PMID: 25786696 PMC4511812

[5] Orkin SH Zon LI . Hematopoiesis: an evolving paradigm for stem cell biology. Cell. (2008) 132:631–44. doi: 10.1016/j.cell.2008.01.025, PMID: 18295580 PMC2628169

[6] Zhao A Zhou H Yang J Li M Niu T . Epigenetic regulation in hematopoiesis and its implications in the targeted therapy of hematologic Malignancies. Signal Transduct Target Ther. (2023) 8:71. doi: 10.1038/s41392-023-01342-6, PMID: 36797244 PMC9935927

[7] Jin B Robertson KD . DNA methyltransferases, DNA damage repair, and cancer. Adv Exp Med Biol. (2013) 754:3–29. 22956494 10.1007/978-1-4419-9967-2_1 PMC3707278

[8] Gao L Emperle M Guo Y Grimm SA Ren W Adam S . Comprehensive structure-function characterization of DNMT3B and DNMT3A reveals distinctive *de novo* DNA methylation mechanisms. Nat Commun. (2020) 11:3355. doi: 10.1038/s41467-020-17109-4, PMID: 32620778 PMC7335073

[9] Zhang YY Yao DM Zhu XW Zhou JD Ma JC Yang J . DNMT3A intragenic hypomethylation is associated with adverse prognosis in acute myeloid leukemia. Leuk Res. (2015) 39:1041–7. doi: 10.1016/j.leukres.2015.06.015, PMID: 26242829

[10] Lujambio A Ropero S Ballestar E Fraga MF Cerrato C Setién F . Genetic unmasking of an epigenetically silenced microRNA in human cancer cells. Cancer Res. (2007) 67:1424–9. doi: 10.1158/0008-5472.CAN-06-4218, PMID: 17308079

[11] Wong KK Lawrie CH Green TM . Oncogenic roles and inhibitors of DNMT1, DNMT3A, and DNMT3B in acute myeloid leukaemia. biomark Insights. (2019) 14:1177271919846454. doi: 10.1177/1177271919846454, PMID: 31105426 PMC6509988

[12] Lopusna K Nowialis P Opavska J Abraham A Riva A Opavsky R . Dnmt3b catalytic activity is critical for its tumour suppressor function in lymphomagenesis and is associated with c-Met oncogenic signalling. EBioMedicine. (2021) 63:3788. doi: 10.1016/j.ebiom.2020.103191, PMID: 33418509 PMC7804970

[13] Ko M An J Pastor WA Koralov SB Rajewsky K Rao A . TET proteins and 5-methylcytosine oxidation in hematological cancers. Immunol Rev. (2015) 263:6–21. doi: 10.1111/imr.2014.263.issue-1 25510268 PMC4617313

[14] Zhang X Zhang Y Wang C Wang X . TET (Ten-eleven translocation) family proteins: structure, biological functions and applications. Signal Transduct Target Ther. (2023) 8:297. doi: 10.1038/s41392-023-01537-x, PMID: 37563110 PMC10415333

[15] Kunimoto H Nakajima H . TET2: A cornerstone in normal and Malignant hematopoiesis. Cancer Sci. (2021) 112:31–40. doi: 10.1111/cas.v112.1, PMID: 33048426 PMC7780023

[16] Ley TJ Ding L Walter MJ McLellan MD Lamprecht T Larson DE . DNMT3A mutations in acute myeloid leukemia. N Engl J Med. (2010) 363:2424–33. doi: 10.1056/NEJMoa1005143, PMID: 21067377 PMC3201818

[17] Stegelmann F Bullinger L Schlenk RF Paschka P Griesshammer M Blersch C . DNMT3A mutations in myeloproliferative neoplasms. Leukemia. (2011) 25:1217–9. doi: 10.1038/leu.2011.77, PMID: 21537334

[18] Jankowska AM Makishima H Tiu RV Szpurka H Huang Y Traina F . Mutational spectrum analysis of chronic myelomonocytic leukemia includes genes associated with epigenetic regulation: UTX, EZH2, and DNMT3A. Blood. (2011) 118:3932–41. doi: 10.1182/blood-2010-10-311019, PMID: 21828135 PMC3193268

[19] Couronné L Bastard C Bernard OA . TET2 and DNMT3A mutations in human T-cell lymphoma. N Engl J Med. (2012) 366:95–6. doi: 10.1056/NEJMc1111708, PMID: 22216861

[20] Grossmann V Haferlach C Weissmann S Roller A Schindela S Poetzinger F . The molecular profile of adult T-cell acute lymphoblastic leukemia: mutations in RUNX1 and DNMT3A are associated with poor prognosis in T-ALL. Genes Chromosomes Cancer. (2013) 52:410–22. doi: 10.1002/gcc.22039, PMID: 23341344

[21] Yang L Rau R Goodell MA . DNMT3A in haematological Malignancies. Nat Rev Cancer. (2015) 15:152–65. doi: 10.1038/nrc3895, PMID: 25693834 PMC5814392

[22] Mayle A Yang L Rodriguez B Zhou T Chang E Curry CV . Dnmt3a loss predisposes murine hematopoietic stem cells to Malignant transformation. Blood. (2015) 125:629–38. doi: 10.1182/blood-2014-08-594648, PMID: 25416277 PMC4304108

[23] Phi LTH Sari IN Yang YG Lee SH Jun N Kim KS . Cancer stem cells (CSCs) in drug resistance and their therapeutic implications in cancer treatment. Stem Cells Int. (2018) 2018:5416923. doi: 10.1155/2018/5416923, PMID: 29681949 PMC5850899

[24] Shlush LI Zandi S Mitchell A Chen WC Brandwein JM Gupta V . Identification of pre-leukaemic haematopoietic stem cells in acute leukaemia. Nature. (2014) 506:328–33. doi: 10.1038/nature13038, PMID: 24522528 PMC4991939

[25] Walker BA Mavrommatis K Wardell CP Ashby TC Bauer M Davies FE . Identification of novel mutational drivers reveals oncogene dependencies in multiple myeloma. Blood. (2018) 132:587–97. doi: 10.1182/blood-2018-03-840132, PMID: 29884741 PMC6097138

[26] Heuck CJ Mehta J Bhagat T Gundabolu K Yu Y Khan S . Myeloma is characterized by stage-specific alterations in DNA methylation that occur early during myelomagenesis. J Immunol. (2013) 190:2966–75. doi: 10.4049/jimmunol.1202493, PMID: 23408834 PMC4581585

[27] Zhang W Xu J . DNA methyltransferases and their roles in tumorigenesis. biomark Res. (2017) 5:1. doi: 10.1186/s40364-017-0081-z, PMID: 28127428 PMC5251331

[28] Shen N Yan F Pang J Zhao N Gangat N Wu L . Inactivation of receptor tyrosine kinases reverts aberrant DNA methylation in acute myeloid leukemia. Clin Cancer Res. (2017) 23:6254–66. doi: 10.1158/1078-0432.CCR-17-0235, PMID: 28720666 PMC5641248

[29] Zhou W Chen H Hong X Niu X Lu Q . Knockdown of DNA methyltransferase-1 inhibits proliferation and derepresses tumor suppressor genes in myeloma cells. Oncol Lett. (2014) 8:2130–4. doi: 10.3892/ol.2014.2481, PMID: 25289094 PMC4186563

[30] Rahmani T Azad M Chahardouli B Nasiri H Vatanmakanian M Kaviani S . Patterns of DNMT1 promoter methylation in patients with acute lymphoblastic leukemia. Int J Hematol Oncol Stem Cell Res. (2017) 11:172–7., PMID: 28989582 PMC5625466

[31] Wang J Hua L Guo M Yang L Liu X Li Y . Nota ble roles of EZH2 and DNMT1 in epigenetic dormancy of the SHP1 gene during the progression of chronic myeloid leukaemia. Oncol Lett. (2017) 13:4979–85. doi: 10.3892/ol.2017.6050, PMID: 28599500 PMC5453028

[32] Shu Y Zhou X Qi X Liu S Li K Tan J . β-Arrestin1 promotes the self-renewal of the leukemia-initiating cell-enriched subpopulation in B-lineage acute lymphoblastic leukemia related to DNMT1 activity. Cancer Lett. (2015) 357:170–8. doi: 10.1016/j.canlet.2014.11.025, PMID: 25444908

[33] Larmonie NSD Arentsen-Peters TCJM Obulkasim A Valerio D Sonneveld E Danen-van Oorschot AA . MN1 overexpression is driven by loss of DNMT3B methylation activity in inv(16) pediatric AML. Oncogene. (2018) 37:107–15. doi: 10.1038/onc.2017.293, PMID: 28892045

[34] Poole CJ Zheng W Lodh A Yevtodiyenko A Liefwalker D Li H . DNMT3B overexpression contributes to aberrant DNA methylation and MYC-driven tumor maintenance in T-ALL and Burkitt’s lymphoma. Oncotarget. (2017) 8:76898–920. doi: 10.18632/oncotarget.20176, PMID: 29100357 PMC5652751

[35] Shah MY Vasanthakumar A Barnes NY Figueroa ME Kamp A Hendrick C . DNMT3B7, a truncated DNMT3B isoform expressed in human tumors, disrupts embryonic development and accelerates lymphomagenesis. Cancer Res. (2010) 70:5840–50. doi: 10.1158/0008-5472.CAN-10-0847, PMID: 20587527 PMC2905468

[36] Lio C-WJ Yuita H Rao A . Dysregulation of the TET family of epigenetic regulators in lymphoid and myeloid Malignancies. Blood. (2019) 134:1487–97. doi: 10.1182/blood.2019791475, PMID: 31467060 PMC6839946

[37] Lasho TL Vallapureddy R Finke CM Mangaonkar A Gangat N Ketterling R . Infrequent occurrence of TET1, TET3, and ASXL2 mutations in myelodysplastic/myeloproliferative neoplasms. Blood Cancer J. (2018) 8:32. doi: 10.1038/s41408-018-0057-8, PMID: 29531217 PMC5849888

[38] Tefferi A Lim KH Abdel-Wahab O Lasho TL Patel J Patnaik MM . Detection of mutant TET2 in myeloid Malignancies other than myeloproliferative neoplasms: CMML, MDS, MDS/MPN and AML. Leukemia. (2009) 23:1343–5. doi: 10.1038/leu.2009.59, PMID: 19295549 PMC4654626

[39] Cimmino L Dawlaty MM Ndiaye-Lobry D Yap YS Bakogianni S Yu Y . TET1 is a tumor suppressor of hematopoietic Malignancy. Nat Immunol. (2015) 16:653–62. doi: 10.1038/ni.3148, PMID: 25867473 PMC4545281

[40] Gao Q Shen K Xiao M . TET2 mutation in acute myeloid leukemia: biology, clinical significance, and therapeutic insights. Clin Epigenetics. (2024) 16:155. doi: 10.1186/s13148-024-01771-2, PMID: 39521964 PMC11550532

[41] Döhner H Weisdorf DJ Bloomfield CD . Acute myeloid leukemia. N Engl J Med. (2015) 373:1136–52. doi: 10.1056/NEJMra1406184, PMID: 26376137

[42] Ferrone CK Blydt-Hansen M Rauh MJ . Age-associated TET2 mutations: common drivers of myeloid dysfunction, cancer and cardiovascular disease. Int J Mol Sci. (2020) 21:626. doi: 10.3390/ijms21020626, PMID: 31963585 PMC7014315

[43] Abdel-Wahab O Mullally A Hedvat C Garcia-Manero G Patel J Wadleigh M . Genetic characterization of TET1, TET2, and TET3 alterations in myeloid Malignancies. Blood. (2009) 114:144–7. doi: 10.1182/blood-2009-03-210039, PMID: 19420352 PMC2710942

[44] Ko M Bandukwala HS An J Lamperti ED Thompson EC Hastie R . Ten-Eleven-Translocation 2 (TET2) negatively regulates homeostasis and differentiation of hematopoietic stem cells in mice. Proc Natl Acad Sci U S A. (2011) 108:14566–71. doi: 10.1073/pnas.1112317108, PMID: 21873190 PMC3167529

[45] Moran-Crusio K Reavie L Shih A Abdel-Wahab O Ndiaye-Lobry D Lobry C . Tet2 loss leads to increased hematopoietic stem cell self-renewal and myeloid transformation. Cancer Cell. (2011) 20:11–24. doi: 10.1016/j.ccr.2011.06.001, PMID: 21723200 PMC3194039

[46] Pan F Wingo TS Zhao Z Gao R Makishima H Qu G . Tet2 loss leads to hypermutagenicity in haematopoietic stem/progenitor cells. Nat Commun. (2017) 8:15102. doi: 10.1038/ncomms15102, PMID: 28440315 PMC5414116

[47] Chow RD Velu P Deihimi S Belman J Youn A Shah N . Persistent postremission clonal hematopoiesis shapes the relapse trajectories of acute myeloid leukemia. Blood Adv. (2025) 9:1888–99. doi: 10.1182/bloodadvances.2024015149, PMID: 39938015 PMC12008691

[48] Ko M Huang Y Jankowska AM Pape UJ Tahiliani M Bandukwala HS . Impaired hydroxylation of 5-methylcytosine in myeloid cancers with mutant TET2. Nature. (2010) 468:839–43. doi: 10.1038/nature09586, PMID: 21057493 PMC3003755

[49] Pronier E Almire C Mokrani H Vasanthakumar A Simon A da Costa Reis Monte Mor B . Inhibition of TET2-mediated conversion of 5-methylcytosine to 5-hydroxymethylcytosine disturbs erythroid and granulomonocytic differentiation of human hematopoietic progenitors. Blood. (2011) 118:2551–5. doi: 10.1182/blood-2010-12-324707, PMID: 21734233 PMC3292425

[50] Quivoron C Couronné L Della Valle V Lopez CK Plo I Wagner-Ballon O . TET2 inactivation results in pleiotropic hematopoietic abnormalities in mouse and is a recurrent event during human lymphomagenesis. Cancer Cell. (2011) 20:25–38. doi: 10.1016/j.ccr.2011.06.003, PMID: 21723201

[51] Kunimoto H Fukuchi Y Sakurai M Takubo K Okamoto S Nakajima H . Tet2-mutated myeloid progenitors possess aberrant *in vitro* self-renewal capacity. Blood. (2014) 123:2897–9. doi: 10.1182/blood-2014-01-552471, PMID: 24786459

[52] Li C Lan Y Schwartz-Orbach L Korol E Tahiliani M Evans T . Overlapping requirements for tet2 and tet3 in normal development and hematopoietic stem cell emergence. Cell Rep. (2015) 12:1133–43. doi: 10.1016/j.celrep.2015.07.025, PMID: 26257178 PMC4545447

[53] López-Moyado IF Tsagaratou A Yuita H Seo H Delatte B Heinz S . Paradoxical association of TET loss of function with genome-wide DNA hypomethylation. Proc Natl Acad Sci U S A. (2019) 116:16933–42. doi: 10.1073/pnas.1903059116, PMID: 31371502 PMC6708373

[54] Figueroa ME Abdel-Wahab O Lu C Ward PS Patel J Shih A . Leukemic IDH1 and IDH2 mutations result in a hypermethylation phenotype, disrupt TET2 function, and impair hematopoietic differentiation. Cancer Cell. (2010) 18:553–67. doi: 10.1016/j.ccr.2010.11.015, PMID: 21130701 PMC4105845

[55] Chowdhury R Yeoh KK Tian YM Hillringhaus L Bagg EA Rose NR . The oncometabolite 2-hydroxyglutarate inhibits histone lysine demethylases. EMBO Rep. (2011) 12:463–9. doi: 10.1038/embor.2011.43, PMID: 21460794 PMC3090014

[56] Medeiros BC Fathi AT DiNardo CD Pollyea DA Chan SM Swords R . Isocitrate dehydrogenase mutations in myeloid Malignancies. Leukemia. (2017) 31:272–81. doi: 10.1038/leu.2016.275, PMID: 27721426 PMC5292675

[57] Rampal R Alkalin A Madzo J Vasanthakumar A Pronier E Patel J . DNA hydroxymethylation profiling reveals that WT1 mutations result in loss of TET2 function in acute myeloid leukemia. Cell Rep. (2014) 9:1841–55. doi: 10.1016/j.celrep.2014.11.004, PMID: 25482556 PMC4267494

[58] Wang C McKeithan TW Gong Q Zhang W Bouska A Rosenwald A . IDH2R172 mutations define a unique subgroup of patients with angioimmunoblastic T-cell lymphoma. Blood. (2015) 126:1741–52. doi: 10.1182/blood-2015-05-644591, PMID: 26268241 PMC4600014

[59] Cairns RA Iqbal J Lemonnier F Kucuk C de Leval L Jais JP . IDH2 mutations are frequent in angioimmunoblastic T-cell lymphoma. Blood. (2012) 119:1901–3. doi: 10.1182/blood-2011-11-391748, PMID: 22215888 PMC3293643

[60] Lemonnier F Cairns RA Inoue S Li WY Dupuy A Broutin S . The IDH2 R172K mutation associated with angioimmunoblastic T-cell lymphoma produces 2HG in T cells and impacts lymphoid development. Proc Natl Acad Sci U S A. (2016) 113:15084–9. doi: 10.1073/pnas.1617929114, PMID: 27956631 PMC5206549

[61] Yao WQ Wu F Zhang W Chuang SS Thompson JS Chen Z . Angioimmunoblastic T-cell lymphoma contains multiple clonal T-cell populations derived from a common TET2 mutant progenitor cell. J Pathol. (2020) 250:346–57. doi: 10.1002/path.v250.3, PMID: 31859368 PMC7064999

[62] Figueroa ME Skrabanek L Li Y Jiemjit A Fandy TE Paietta E . MDS and secondary AML display unique patterns and abundance of aberrant DNA methylation. Blood. (2009) 114:3448–58. doi: 10.1182/blood-2009-01-200519, PMID: 19652201 PMC2765680

[63] Jiang Y Dunbar A Gondek LP Mohan S Rataul M O’Keefe C . Aberrant DNA methylation is a dominant mechanism in MDS progression to AML. Blood. (2009) 113:1315–25. doi: 10.1182/blood-2008-06-163246, PMID: 18832655 PMC2637194

[64] Giacopelli B Wang M Cleary A Wu YZ Schultz AR Schmutz M . DNA methylation epitypes highlight underlying developmental and disease pathways in acute myeloid leukemia. Genome Res. (2021) 31:747–61. doi: 10.1101/gr.269233.120, PMID: 33707228 PMC8092005

[65] Kelly AD Kroeger H Yamazaki J Taby R Neumann F Yu S . A CpG island methylator phenotype in acute myeloid leukemia independent of IDH mutations and associated with a favorable outcome. Leukemia. (2017) 31:2011–9. doi: 10.1038/leu.2017.12, PMID: 28074068 PMC5537054

[66] Šestáková Š Šálek C Kundrát D Cerovská E Vydra J Ježíšková I . MethScore as a new comprehensive DNA methylation-based value refining the prognosis in acute myeloid leukemia. Clin Epigenetics. (2024) 16:17. doi: 10.1186/s13148-024-01625-x, PMID: 38254139 PMC10802002

[67] Espín-Pérez A Brennan K Ediriwickrema AS Gevaert O Lossos IS Gentles AJ . Peripheral blood DNA methylation profiles predict future development of B-cell Non-Hodgkin Lymphoma. NPJ Precis Oncol. (2022) 6:53. doi: 10.1038/s41698-022-00295-3, PMID: 35864305 PMC9304422

[68] Muylaert C Van Hemelrijck LA Maes A De Veirman K Menu E Vanderkerken K . Aberrant DNA methylation in multiple myeloma: A major obstacle or an opportunity? Front Oncol. (2022) 12:979569. doi: 10.3389/fonc.2022.979569, PMID: 36059621 PMC9434119

[69] Agirre X Castellano G Pascual M Heath S Kulis M Segura V . Whole-epigenome analysis in multiple myeloma reveals DNA hypermethylation of B cell-specific enhancers. Genome Res. (2015) 25:478–87. doi: 10.1101/gr.180240.114, PMID: 25644835 PMC4381520

[70] Ng MH Chung YF Lo KW Wickham NW Lee JC Huang DP . Frequent hypermethylation of p16 and p15 genes in multiple myeloma. Blood. (1997) 89:2500–6. doi: 10.1182/blood.V89.7.2500 9116295

[71] Chim CS Pang R Fung TK Choi CL Liang R . Epigenetic dysregulation of Wnt signaling pathway in multiple myeloma. Leukemia. (2007) 21:2527–36. doi: 10.1038/sj.leu.2404939, PMID: 17882284

[72] van Andel H Kocemba KA Spaargaren M Pals ST . Aberrant Wnt signaling in multiple myeloma: molecular mechanisms and targeting options. Leukemia. (2019) 33:1063–75. doi: 10.1038/s41375-019-0404-1, PMID: 30770859 PMC6756057

[73] Turner JG Gump JL Zhang C Cook JM Marchion D Hazlehurst L . ABCG2 expression, function, and promoter methylation in human multiple myeloma. Blood. (2006) 108:3881–9. doi: 10.1182/blood-2005-10-009084, PMID: 16917002 PMC1895461

[74] Garcia-Manero G Yang H Kuang SQ O’Brien S Thomas D Kantarjian H . Epigenetics of acute lymphocytic leukemia. Semin Hematol. (2009) 46:24–32. doi: 10.1053/j.seminhematol.2008.09.008, PMID: 19100365 PMC3833728

[75] Wahlberg P Lundmark A Nordlund J Busche S Raine A Tandre K . DNA methylome analysis of acute lymphoblastic leukemia cells reveals stochastic *de novo* DNA methylation in CpG islands. Epigenomics. (2016) 8:1367–87. doi: 10.2217/epi-2016-0052, PMID: 27552300

[76] Yosifov DY Bloehdorn J Döhner H Lichter P Stilgenbauer S Mertens D . DNA methylation of chronic lymphocytic leukemia with differential response to chemotherapy. Sci Data. (2020) 7:133. doi: 10.1038/s41597-020-0456-0, PMID: 32358561 PMC7195470

[77] Bu S Ye T Gao H Song H Zhu Y . Histone methylation and acetylation in cancer: mechanism, progression, and targets. Oncologie. (2025) 27:29–43. doi: 10.1515/oncologie-2024-0324

[78] Cao J Yan Q . Histone ubiquitination and deubiquitination in transcription, DNA damage response, and cancer. Front Oncol. (2012) 2:26. doi: 10.3389/fonc.2012.00026, PMID: 22649782 PMC3355875

[79] Strahl BD Allis CD . The language of covalent histone modifications. Nature. (2000) 403:41–5. doi: 10.1038/47412, PMID: 10638745

[80] Hyun K Jeon J Park K Kim J . Writing, erasing and reading histone lysine methylations. Exp Mol Med. (2017) 49:e324. doi: 10.1038/emm.2017.11, PMID: 28450737 PMC6130214

[81] Canaani E Nakamura T Rozovskaia T Smith ST Mori T Croce CM . ALL-1/MLL1, a homologue of Drosophila TRITHORAX, modifies chromatin and is directly involved in infant acute leukaemia. Br J Cancer. (2004) 90:756–60. doi: 10.1038/sj.bjc.6601639, PMID: 14970849 PMC2410188

[82] Li BE Ernst P . Two decades of leukemia oncoprotein epistasis: the MLL1 paradigm for epigenetic deregulation in leukemia. Exp Hematol. (2014) 42:995–1012. doi: 10.1016/j.exphem.2014.09.006, PMID: 25264566 PMC4307938

[83] Chen Y Ernst P . Hematopoietic transformation in the absence of MLL1/KMT2A: distinctions in target gene reactivation. Cell Cycle. (2019) 18:1525–31. doi: 10.1080/15384101.2019.1618642, PMID: 31161857 PMC6619971

[84] Nakamura T Mori T Tada S Krajewski W Rozovskaia T Wassell R . ALL-1 is a histone methyltransferase that assembles a supercomplex of proteins involved in transcriptional regulation. Mol Cell. (2002) 10:1119–28. doi: 10.1016/S1097-2765(02)00740-2, PMID: 12453419

[85] Collins CT Hess JL . Role of HOXA9 in leukemia: dysregulation, cofactors and essential targets. Oncogene. (2016) 35:1090–8. doi: 10.1038/onc.2015.174, PMID: 26028034 PMC4666810

[86] Goto H Suenobu S Koga Y Yamamoto S Nakashima K Oba U . H3K27me3 and HOXA9 expression predict prognosis in pediatric acute myeloid leukemia: an epigenetic-transcriptional correlation study. Front Hematol. (2025) 4:1668408. doi: 10.3389/frhem.2025.1668408

[87] Sun Y Zhou B Mao F Xu J Miao H Zou Z . HOXA9 reprograms the enhancer landscape to promote leukemogenesis. Cancer Cell. (2018) 34:643–58.e5. doi: 10.1016/j.ccell.2018.08.018, PMID: 30270123 PMC6179449

[88] Zehtabcheh S Soleimani Samarkhazan H Asadi M Zabihi M Parkhideh S Mohammadi MH . Insights into KMT2A rearrangements in acute myeloid leukemia: from molecular characteristics to targeted therapies. biomark Res. (2025) 13:73. doi: 10.1186/s40364-025-00786-y, PMID: 40361241 PMC12077025

[89] Fang J Zhang J Zhu L Xin X Hu H . The epigenetic role of EZH2 in acute myeloid leukemia. PeerJ. (2024) 12:e18656. doi: 10.7717/peerj.18656, PMID: 39655332 PMC11627098

[90] Pawlyn C Bright MD Buros AF Stein CK Walters Z Aronson LI . Overexpression of EZH2 in multiple myeloma is associated with poor prognosis and dysregulation of cell cycle control. Blood Cancer J. (2017) 7:e549. doi: 10.1038/bcj.2017.27, PMID: 28362441 PMC5380911

[91] Tanaka S Miyagi S Sashida G Chiba T Yuan J Mochizuki-Kashio M . Ezh2 augments leukemogenicity by reinforcing differentiation blockage in acute myeloid leukemia. Blood. (2012) 120:1107–17. doi: 10.1182/blood-2011-11-394932, PMID: 22677129

[92] Kempf JM Weser S Bartoschek MD Metzeler KH Vick B Herold T . Loss-of-function mutations in the histone methyltransferase EZH2 promote chemotherapy resistance in AML. Sci Rep. (2021) 11:5838. doi: 10.1038/s41598-021-84708-6, PMID: 33712646 PMC7955088

[93] Xie H Peng C Huang J Li BE Kim W Smith EC . Chronic myelogenous leukemia- initiating cells require polycomb group protein EZH2. Cancer Discov. (2016) 6:1237–47. doi: 10.1158/2159-8290.CD-15-1439, PMID: 27630126 PMC5096974

[94] Xu L Wang Y Wang G Guo S Yu D Feng Q . Aberrant activation of TRIP13-EZH2 signaling axis promotes stemness and therapy resistance in multiple myeloma. Leukemia. (2023) 37:1576–9. doi: 10.1038/s41375-023-01925-w, PMID: 37157015

[95] van Kemenade FJ Raaphorst FM Blokzijl T Fieret E Hamer KM Satijn DP . Coexpression of BMI-1 and EZH2 polycomb-group proteins is associated with cycling cells and degree of Malignancy in B-cell non-Hodgkin lymphoma. Blood. (2001) 97:3896–901. doi: 10.1182/blood.V97.12.3896, PMID: 11389032

[96] Yan J Ng SB Tay JL Lin B Koh TL Tan J . EZH2 overexpression in natural killer/T-cell lymphoma confers growth advantage independently of histone methyltransferase activity. Blood. (2013) 121:4512–20. doi: 10.1182/blood-2012-08-450494, PMID: 23529930

[97] Huet S Xerri L Tesson B Mareschal S Taix S Mescam-Mancini L . EZH2 alterations in follicular lymphoma: biological and clinical correlations. Blood Cancer J. (2017) 7:e555. doi: 10.1038/bcj.2017.32, PMID: 28430172 PMC5436075

[98] Morin RD Johnson NA Severson TM Mungall AJ An J Goya R . Somatic mutations altering EZH2 (Tyr641) in follicular and diffuse large B-cell lymphomas of germinal-center origin. Nat Genet. (2010) 42:181–5. doi: 10.1038/ng.518, PMID: 20081860 PMC2850970

[99] Ernst T Chase AJ Score J Hidalgo-Curtis CE Bryant C Jones AV . Inactivating mutations of the histone methyltransferase gene EZH2 in myeloid disorders. Nat Genet. (2010) 42:722–6. doi: 10.1038/ng.621, PMID: 20601953

[100] Hernando H Gelato KA Lesche R Beckmann G Koehr S Otto S . EZH2 inhibition blocks multiple myeloma cell growth through upregulation of epithelial tumor suppressor genes. Mol Cancer Ther. (2016) 15:287–98. doi: 10.1158/1535-7163.MCT-15-0486, PMID: 26590165

[101] Chemlal D Varlet E Machura A Ovejero S Requirand G Robert N . EZH2 targeting induces CD38 upregulation and response to anti-CD38 immunotherapies in multiple myeloma. Leukemia. (2023) 37:1925–8. doi: 10.1038/s41375-023-01983-0, PMID: 37532787 PMC10457196

[102] Straining R Eighmy W . Tazemetostat: EZH2 inhibitor. J Adv Pract Oncol. (2022) 13:158–63. doi: 10.6004/jadpro.2022.13.2.7, PMID: 35369397 PMC8955562

[103] Johnson WL Yewdell WT Bell JC McNulty SM Duda Z O’Neill RJ . RNA-dependent stabilization of SUV39H1 at constitutive heterochromatin. Elife. (2017) 6:e25299. doi: 10.7554/eLife.25299, PMID: 28760200 PMC5538822

[104] Chu Y Chen Y Guo H Li M Wang B Shi D . SUV39H1 regulates the progression of MLL-AF9-induced acute myeloid leukemia. Oncogene. (2020) 39:7239–52. doi: 10.1038/s41388-020-01495-6, PMID: 33037410 PMC7728597

[105] Monaghan L Massett ME Bunschoten RP Hoose A Pirvan PA Liskamp RMJ . The emerging role of H3K9me3 as a potential therapeutic target in acute myeloid leukemia. Front Oncol. (2019) 9:705. doi: 10.3389/fonc.2019.00705, PMID: 31428579 PMC6687838

[106] Müller-Tidow C Klein HU Hascher A Isken F Tickenbrock L Thoennissen N . Profiling of histone H3 lysine 9 trimethylation levels predicts transcription factor activity and survival in acute myeloid leukemia. Blood. (2010) 116:3564–71. doi: 10.1182/blood-2009-09-240978, PMID: 20498303 PMC2981478

[107] Lakshmikuttyamma A Scott SA DeCoteau JF Geyer CR . Reexpression of epigenetically silenced AML tumor suppressor genes by SUV39H1 inhibition. Oncogene. (2010) 29:576–88. doi: 10.1038/onc.2009.361, PMID: 19881540

[108] Martinez-Garcia E Popovic R Min DJ Sweet SM Thomas PM Zamdborg L . The MMSET histone methyl transferase switches global histone methylation and alters gene expression in t (4,14) multiple myeloma cells. Blood. (2011) 117:211–20. doi: 10.1182/blood-2010-07-298349, PMID: 20974671 PMC3037745

[109] Keats JJ Reiman T Maxwell CA Taylor BJ Larratt LM Mant MJ . In multiple myeloma, t (4,14)(p16;q32) is an adverse prognostic factor irrespective of FGFR3 expression. Blood. (2003) 101:1520–9. doi: 10.1182/blood-2002-06-1675, PMID: 12393535

[110] Shah MY Martinez-Garcia E Phillip JM Chambliss AB Popovic R Ezponda T . MMSET/WHSC1 enhances DNA damage repair leading to an increase in resistance to chemotherapeutic agents. Oncogene. (2016) 35:5905–15. doi: 10.1038/onc.2016.116, PMID: 27109101 PMC6071667

[111] Lauring J Abukhdeir AM Konishi H Garay JP Gustin JP Wang Q . The multiple myeloma associated MMSET gene contributes to cellular adhesion, clonogenic growth, and tumorigenicity. Blood. (2008) 111:856–64. doi: 10.1182/blood-2007-05-088674, PMID: 17942756 PMC2200833

[112] Popovic R Martinez-Garcia E Giannopoulou EG Zhang Q Zhang Q Ezponda T . Histone methyltransferase MMSET/NSD2 alters EZH2 binding and reprograms the myeloma epigenome through global and focal changes in H3K36 and H3K27 methylation. PloS Genet. (2014) 10:e1004566. doi: 10.1371/journal.pgen.1004566, PMID: 25188243 PMC4154646

[113] Magliulo D Bernardi R Messina S . Lysine-specific demethylase 1A as a promising target in acute myeloid leukemia. Front Oncol. (2018) 8:255. doi: 10.3389/fonc.2018.00255, PMID: 30073149 PMC6060236

[114] Fang J Ying H Mao T Fang Y Lu Y Wang H . Upregulation of CD11b and CD86 through LSD1 inhibition promotes myeloid differentiation and suppresses cell proliferation in human monocytic leukemia cells. Oncotarget. (2017) 8:85085–101. doi: 10.18632/oncotarget.18564, PMID: 29156705 PMC5689595

[115] Harris WJ Huang X Lynch JT Spencer GJ Hitchin JR Li Y . The histone demethylase KDM1A sustains the oncogenic potential of MLL-AF9 leukemia stem cells. Cancer Cell. (2012) 21:473–87. doi: 10.1016/j.ccr.2012.03.014, PMID: 22464800

[116] Xu S Li X Zhang J Chen J . Prognostic value of CD11b expression level for acute myeloid leukemia patients: A meta-analysis. PloS One. (2015) 10:e0135981. doi: 10.1371/journal.pone.0135981, PMID: 26309131 PMC4550244

[117] Graf M Reif S Kröll T Hecht K Nuessler V Schmetzer H . Expression of MAC-1 (CD11b) in acute myeloid leukemia (AML) is associated with an unfavorable prognosis. Am J Hematol. (2006) 81:227–35. doi: 10.1002/ajh.20526, PMID: 16550517

[118] Zhang Q Ma R Chen H Guo W Li Z Xu K . CD86 is associated with immune infiltration and immunotherapy signatures in AML and promotes its progression. J Oncol. (2023) 2023:9988405. doi: 10.1155/2023/9988405, PMID: 37064861 PMC10104747

[119] Niebel D Kirfel J Janzen V Höller T Majores M Gütgemann I . Lysine-specific demethylase 1 (LSD1) in hematopoietic and lymphoid neoplasms. Blood. (2014) 124:151–2. doi: 10.1182/blood-2014-04-569525, PMID: 24993879

[120] González-Novo R Armesto M González-Murillo Á Dreger M Hurlstone AFL Benito A . Dual effect of targeting LSD1 on the invasiveness and the mechanical response of acute lymphoblastic leukemia cells. BioMed Pharmacother. (2025) 183:117830. doi: 10.1016/j.biopha.2025.117830, PMID: 39818101

[121] Wang P Wang Z Liu J . Role of HDACs in normal and Malignant hematopoiesis. Mol Cancer. (2020) 19:5. doi: 10.1186/s12943-019-1127-7, PMID: 31910827 PMC6945581

[122] Li L Zeng Y Cheng G Yang H . Acetylation and deacetylation dynamics in stress response to cancer and infections. Semin Immunol. (2025) 78:101957. doi: 10.1016/j.smim.2025.101957, PMID: 40288003

[123] Mullighan CG Zhang J Kasper LH Lerach S Payne-Turner D Phillips LA . CREBBP mutations in relapsed acute lymphoblastic leukaemia. Nature. (2011) 471:235–9. doi: 10.1038/nature09727, PMID: 21390130 PMC3076610

[124] Pasqualucci L Dominguez-Sola D Chiarenza A Fabbri G Grunn A Trifonov V . Inactivating mutations of acetyltransferase genes in B-cell lymphoma. Nature. (2011) 471:189–95. doi: 10.1038/nature09730, PMID: 21390126 PMC3271441

[125] Gao XN Lin J Ning QY Gao L Yao YS Zhou JH . A histone acetyltransferase p300 inhibitor C646 induces cell cycle arrest and apoptosis selectively in AML1-ETO-positive AML cells. PloS One. (2013) 8:e55481. doi: 10.1371/journal.pone.0055481, PMID: 23390536 PMC3563640

[126] Huang Y Chen J Lu C Han J Wang G Song C . HDAC1 and Klf4 interplay critically regulates human myeloid leukemia cell proliferation. Cell Death Dis. (2014) 5:e1491. doi: 10.1038/cddis.2014.433, PMID: 25341045 PMC4237257

[127] Grignani F De Matteis S Nervi C Tomassoni L Gelmetti V Cioce M . Fusion proteins of the retinoic acid receptor-alpha recruit histone deacetylase in promyelocytic leukaemia. Nature. (1998) 391:815–8. doi: 10.1038/35901, PMID: 9486655

[128] Gelmetti V Zhang J Fanelli M Minucci S Pelicci PG Lazar MA . Aberrant recruitment of the nuclear receptor corepressor-histone deacetylase complex by the acute myeloid leukemia fusion partner ETO. Mol Cell Biol. (1998) 18:7185–91. doi: 10.1128/MCB.18.12.7185, PMID: 9819405 PMC109300

[129] Lai TH Ozer HG Gasparini P Nigita G Distefano R Yu L . HDAC1 regulates the chromatin landscape to control transcriptional dependencies in chronic lymphocytic leukemia. Blood Adv. (2023) 7:2897–911. doi: 10.1182/bloodadvances.2022007998, PMID: 36287107 PMC10285551

[130] Bamodu OA Kuo KT Yuan LP Cheng WH Lee WH Ho YS . HDAC inhibitor suppresses proliferation and tumorigenicity of drug-resistant chronic myeloid leukemia stem cells through regulation of hsa-miR-196a targeting BCR/ABL1. Exp Cell Res. (2018) 370:519–30. doi: 10.1016/j.yexcr.2018.07.017, PMID: 30017934

[131] Chen IC Sethy B Liou JP . Recent update of HDAC inhibitors in lymphoma. Front Cell Dev Biol. (2020) 8:576391. doi: 10.3389/fcell.2020.576391, PMID: 33015069 PMC7494784

[132] Mithraprabhu S Kalff A Chow A Khong T Spencer A . Dysregulated Class I histone deacetylases are indicators of poor prognosis in multiple myeloma. Epigenetics. (2014) 9:1511–20. doi: 10.4161/15592294.2014.983367, PMID: 25482492 PMC4622977

[133] El Omari N Bakrim S Elhrech H Aanniz T Balahbib A Lee LH . Clinical efficacy and mechanistic insights of FDA-approved HDAC inhibitors in the treatment of lymphoma. Eur J Pharm Sci. (2025) 208:107057. doi: 10.1016/j.ejps.2025.107057, PMID: 40043823

[134] Clapier CR Iwasa J Cairns BR Peterson CL . Mechanisms of action and regulation of ATP-dependent chromatin-remodelling complexes. Nat Rev Mol Cell Biol. (2017) 18:407–22. doi: 10.1038/nrm.2017.26, PMID: 28512350 PMC8127953

[135] Andrades A Peinado P Alvarez-Perez JC Sanjuan-Hidalgo J García DJ Arenas AM . SWI/SNF complexes in hematological Malignancies: biological implications and therapeutic opportunities. Mol Cancer. (2023) 22:39. doi: 10.1186/s12943-023-01736-8, PMID: 36810086 PMC9942420

[136] Saha S Samal P Madhulika S Murmu KC Chakraborty S Basu J . SMARCD1 negatively regulates myeloid differentiation of leukemic cells via epigenetic mechanisms. Blood Adv. (2022) 6:3106–13. doi: 10.1182/bloodadvances.2021006235, PMID: 35078226 PMC9131909

[137] Madan V Shyamsunder P Han L Mayakonda A Nagata Y Sundaresan J . Comprehensive mutational analysis of primary and relapse acute promyelocytic leukemia. Leukemia. (2016) 30:1672–81. doi: 10.1038/leu.2016.69, PMID: 27063598 PMC4972641

[138] Barisic D Chin CR Meydan C Teater M Tsialta I Mlynarczyk C . ARID1A orchestrates SWI/SNF-mediated sequential binding of transcription factors with ARID1A loss driving pre-memory B cell fate and lymphomagenesis. Cancer Cell. (2024) 42:583–604.e11. 38458187 10.1016/j.ccell.2024.02.010 PMC11407687

[139] Korfi K Ali S Heward JA Fitzgibbon J . Follicular lymphoma, a B cell Malignancy addicted to epigenetic mutations. Epigenetics. (2017) 12:370–7. doi: 10.1080/15592294.2017.1282587, PMID: 28106467 PMC5453190

[140] Chakraborty C Talluri S Binder M Morelli E Mayoral JE Derebail S . Loss of BCL7A permits IRF4 transcriptional activity and cellular growth in multiple myeloma. Blood. (2025) 146:104–14. doi: 10.1182/blood.2024026588, PMID: 40090008 PMC12782957

[141] Gutierrez A Kentsis A Sanda T Holmfeldt L Chen SC Zhang J . The BCL11B tumor suppressor is mutated across the major molecular subtypes of T-cell acute lymphoblastic leukemia. Blood. (2011) 118:4169–73. doi: 10.1182/blood-2010-11-318873, PMID: 21878675 PMC3204734

[142] Turkova T Kokavec J Zikmund T Dibus N Pimkova K Nemec D . Differential requirements for Smarca5 expression during hematopoietic stem cell commitment. Commun Biol. (2024) 7:244. doi: 10.1038/s42003-024-05917-z, PMID: 38424235 PMC10904812

[143] Boeger H Griesenbeck J Strattan JS Kornberg RD . Removal of promoter nucleosomes by disassembly rather than sliding *in vivo*. Mol Cell. (2004) 14:667–73. doi: 10.1016/j.molcel.2004.05.013, PMID: 15175161

[144] Aydin ÖZ Vermeulen W Lans H . ISWI chromatin remodeling complexes in the DNA damage response. Cell Cycle. (2014) 13:3016–25. doi: 10.4161/15384101.2014.956551, PMID: 25486562 PMC4615051

[145] Lieleg C Ketterer P Nuebler J Ludwigsen J Gerland U Dietz H . Nucleosome spacing generated by ISWI and CHD1 remodelers is constant regardless of nucleosome density. Mol Cell Biol. (2015) 35:1588–605. doi: 10.1128/MCB.01070-14, PMID: 25733687 PMC4387221

[146] Li Y Gong H Wang P Zhu Y Peng H Cui Y . The emerging role of ISWI chromatin remodeling complexes in cancer. J Exp Clin Cancer Res. (2021) 40:346. doi: 10.1186/s13046-021-02151-x, PMID: 34736517 PMC8567610

[147] Stopka T Zakova D Fuchs O Kubrova O Blafkova J Jelinek J . Chromatin remodeling gene SMARCA5 is dysregulated in primitive hematopoietic cells of acute leukemia. Leukemia. (2000) 14:1247–52. doi: 10.1038/sj.leu.2401807, PMID: 10914549

[148] Dluhosova M Curik N Vargova J Jonasova A Zikmund T Stopka T . Epigenetic control of SPI1 gene by CTCF and ISWI ATPase SMARCA5. PloS One. (2014) 9:e87448. doi: 10.1371/journal.pone.0087448, PMID: 24498324 PMC3911986

[149] Zikmund T Paszekova H Kokavec J Kerbs P Thakur S Turkova T . Loss of ISWI ATPase SMARCA5 (SNF2H) in acute myeloid leukemia cells inhibits proliferation and chromatid cohesion. Int J Mol Sci. (2020) 21(6):2073. doi: 10.3390/ijms21062073, PMID: 32197313 PMC7139293

[150] Hanlon K Rudin CE Harries LW . Investigating the targets of MIR-15a and MIR-16–1 in patients with chronic lymphocytic leukemia (CLL). PloS One. (2009) 4:e7169. doi: 10.1371/journal.pone.0007169, PMID: 19779621 PMC2745703

[151] Alendar A Berns A . Sentinels of chromatin: chromodomain helicase DNA-binding proteins in development and disease. . Genes Dev. (2021) 35:1403–30. doi: 10.1101/gad.348897.121, PMID: 34725129 PMC8559672

[152] Lu Y Jiang J He Z Bao Z Chen X Cheng J . Molecular characteristics and oncogenic role of CHD family genes: a pan-cancer analysis based on bioinformatic and biological analysis. Sci Rep. (2024) 14:18923. doi: 10.1038/s41598-024-68644-9, PMID: 39143142 PMC11324730

[153] Sperlazza J Rahmani M Beckta J Aust M Hawkins E Wang SZ . Depletion of the chromatin remodeler CHD4 sensitizes AML blasts to genotoxic agents and reduces tumor formation. Blood. (2015) 126:1462–72. doi: 10.1182/blood-2015-03-631606, PMID: 26265695 PMC4573869

[154] Rodríguez D Bretones G Quesada V Villamor N Arango JR López-Guillermo A . Mutations in CHD2 cause defective association with active chromatin in chronic lymphocytic leukemia. Blood. (2015) 126:195–202. doi: 10.1182/blood-2014-10-604959, PMID: 26031915

[155] Xiong S Yan Q Peng Y Huang S Zhao R . Overexpression of chromodomain helicase DNA binding protein 5 (CHD5) inhibits cell proliferation and induces cell cycle arrest and apoptosis in chronic myeloid leukemia. Transl Cancer Res. (2021) 10:768–78. doi: 10.21037/tcr-20-2276, PMID: 35116408 PMC8797277

[156] Xu X He Y Miao X Wu Y Han J Wang Q . Cell adhesion induces overexpression of chromodomain helicase/ATPase DNA binding protein 1-like gene (CHD1L) and contributes to cell adhesion-mediated drug resistance (CAM-DR) in multiple myeloma cells. Leuk Res. (2016) 47:54–62. doi: 10.1016/j.leukres.2016.05.007, PMID: 27258734

[157] Brahma S Udugama MI Kim J Hada A Bhardwaj SK Hailu SG . INO80 exchanges H2A.Z for H2A by translocating on DNA proximal to histone dimers. Nat Commun. (2017) 8:15616. doi: 10.1038/ncomms15616, PMID: 28604691 PMC5472786

[158] Giaimo BD Ferrante F Herchenröther A Hake SB Borggrefe T . The histone variant H2A.Z in gene regulation. Epigenet Chromatin. (2019) 12:37. doi: 10.1186/s13072-019-0274-9, PMID: 31200754 PMC6570943

[159] Ávila-López PA Guerrero G Nuñez-Martínez HN Peralta-Alvarez CA Hernández-Montes G Álvarez-Hilario LG . H2A.Z overexpression suppresses senescence and chemosensitivity in pancreatic ductal adenocarcinoma. Oncogene. (2021) 40:2065–80. doi: 10.1038/s41388-021-01664-1, PMID: 33627784 PMC7979544

[160] Vardabasso C Gaspar-Maia A Hasson D Pünzeler S Valle-Garcia D Straub T . Histone variant H2A. Z.2 Mediates Proliferation Drug Sensitivity Malignant Melanoma. Mol Cell. (2015) 59:75–88. doi: 10.1016/j.molcel.2015.05.009, PMID: 26051178 PMC4490946

[161] Dryhurst D Ausió J . Histone H2A.Z deregulation in prostate cancer. Cause or effect? Cancer Metastasis Rev. (2014) 33:429–39. 10.1007/s10555-013-9486-9 PMC4113680 24398858

[162] Bakhshi TJ Georgel PT . Genetic and epigenetic determinants of diffuse large B-cell lymphoma. Blood Cancer J. (2020) 10:123. doi: 10.1038/s41408-020-00389-w, PMID: 33277464 PMC7718920

[163] Dan J Du Z Zhang J Chen T . The interplay between H3K36 methylation and DNA methylation in cancer. Cancer Biol Med. (2023) 20:545–52. doi: 10.20892/j.issn.2095-3941.2023.0234, PMID: 37602556 PMC10476472

[164] El-Osta A Wolffe AP . DNA methylation and histone deacetylation in the control of gene expression: basic biochemistry to human development and disease. Gene Expr. (2000) 9:63–75. doi: 10.3727/000000001783992731, PMID: 11097425 PMC5964960

[165] Kim JY Kim KB Eom GH Choe N Kee HJ Son HJ . KDM3B is the H3K9 demethylase involved in transcriptional activation of lmo2 in leukemia. Mol Cell Biol. (2012) 32:2917–33. doi: 10.1128/MCB.00133-12, PMID: 22615488 PMC3416203

[166] Heuser M Yun H Thol F . Epigenetics in myelodysplastic syndromes. Semin Cancer Biol. (2018) 51:170–9. doi: 10.1016/j.semcancer.2017.07.009, PMID: 28778402 PMC7116652

[167] Dai W Qiao X Fang Y Guo R Bai P Liu S . Epigenetics-targeted drugs: current paradigms and future challenges. Signal Transduct Target Ther. (2024) 9:332. doi: 10.1038/s41392-024-02039-0, PMID: 39592582 PMC11627502

[168] Derissen EJ Beijnen JH Schellens JH . Concise drug review: azacitidine and decitabine. Oncologist. (2013) 18:619–24. doi: 10.1634/theoncologist.2012-0465, PMID: 23671007 PMC3662854

[169] Stresemann C Bokelmann I Mahlknecht U Lyko F . Azacytidine causes complex DNA methylation responses in myeloid leukemia. Mol Cancer Ther. (2008) 7:2998–3005. doi: 10.1158/1535-7163.MCT-08-0411, PMID: 18790780

[170] Stomper J Rotondo JC Greve G Lübbert M . Hypomethylating agents (HMA) for the treatment of acute myeloid leukemia and myelodysplastic syndromes: mechanisms of resistance and novel HMA-based therapies. Leukemia. (2021) 35:1873–89. doi: 10.1038/s41375-021-01218-0, PMID: 33958699 PMC8257497

[171] Kim HJ Bae SC . Histone deacetylase inhibitors: molecular mechanisms of action and clinical trials as anti-cancer drugs. Am J Transl Res. (2011) 3:166–79., PMID: 21416059 PMC3056563

[172] Yang L Qiu Q Wang J Wen Y Li H Liang R . Preclinical and first-in-human of purinostat mesylate, a novel selective HDAC I/IIb inhibitor, in relapsed/refractory multiple myeloma and lymphoma. Signal Transduct Target Ther. (2025) 10:201. doi: 10.1038/s41392-025-02285-w, PMID: 40562746 PMC12198407

[173] Lee JH Choy ML Marks PA . Mechanisms of resistance to histone deacetylase inhibitors. Adv Cancer Res. (2012) 116:39–86. 23088868 10.1016/B978-0-12-394387-3.00002-1

[174] Fruchtman H Avigan ZM Waksal JA Brennan N Mascarenhas JO . Management of isocitrate dehydrogenase 1/2 mutated acute myeloid leukemia. Leukemia. (2024) 38:927–35. doi: 10.1038/s41375-024-02246-2, PMID: 38600315 PMC11073971

[175] Liu X Gong Y . Isocitrate dehydrogenase inhibitors in acute myeloid leukemia. biomark Res. (2019) 7:22. doi: 10.1186/s40364-019-0173-z, PMID: 31660152 PMC6806510

[176] Hosseini A Dhall A Ikonen N Sikora N Nguyen S Shen Y . Perturbing LSD1 and WNT rewires transcription to synergistically induce AML differentiation. Nature. (2025) 642:508–18. doi: 10.1038/s41586-025-08915-1, PMID: 40240608 PMC12158781

